# Development of AIE active carbazole based fluorescent probe for rapid detection of 4-nitrotoluene in solution and solid phases: experimental and DFT insights

**DOI:** 10.1039/d6ra08681k

**Published:** 2026-11-04

**Authors:** Saleh S. Alarfaji, Alam Shabbir, Huda Khalid, Qudsia Sajid, Asad Muhammad Khan, Umar Farooq, Sohail Anjum Shahzad

**Affiliations:** a Department of Chemistry, Faculty of Science, King Khalid University P.O. Box 960 Abha 61421 Saudi Arabia; b Department of Chemistry, COMSATS University Islamabad Abbottabad Campus, University Road Abbottabad 22060 Pakistan sashahzad@cuiatd.edu.pk; c School of Mathematical and Physical Sciences, Faculty of Science, University of Technology Sydney Sydney New South Wales Australia

## Abstract

A fluorescent probe CPT has been strategically designed and synthesized *via* Ullmann and Claisen–Schmidt condensation reactions, and its photophysical properties were systematically investigated. Aggregation-induced emission (AIE) with a 25 nm hypsochromic shift, was attributed to aggregate formation. Further, probe CPT exhibited distinct solvatochromic behavior with a 31 nm bathochromic shift. Owing to these unique optical properties, probe CPT serves as an efficient sensing tool for 4-nitrotoluene (4-NT), exhibiting a fluorescence-quenching response *via* a twisted intramolecular charge transfer (TICT) and photoinduced electron transfer (PET) mechanism. Probe CPT exhibited a hypsochromic shift of 39 nm upon treating with 4-NT. The interaction mechanism was investigated using ^1^H NMR, FTIR, mass spectrometry titration, DLS, and DFT calculations. CPT exhibited excellent selectivity and high sensitivity toward 4-NT, with an LOD of 0.16 nM and an LOQ of 0.55 nM. Furthermore, probe CPT exhibited remarkable efficacy in solid and solution-phase sensing of 4-NT, highlighting its potential applicability for monitoring 4-NT in real industrial effluents.

## Abstract 2

A fluorescent probe CPT has been strategically designed and synthesized *via* Ullmann and Claisen–Schmidt condensation reactions, and its photophysical properties were systematically investigated.

## Graphical abstract

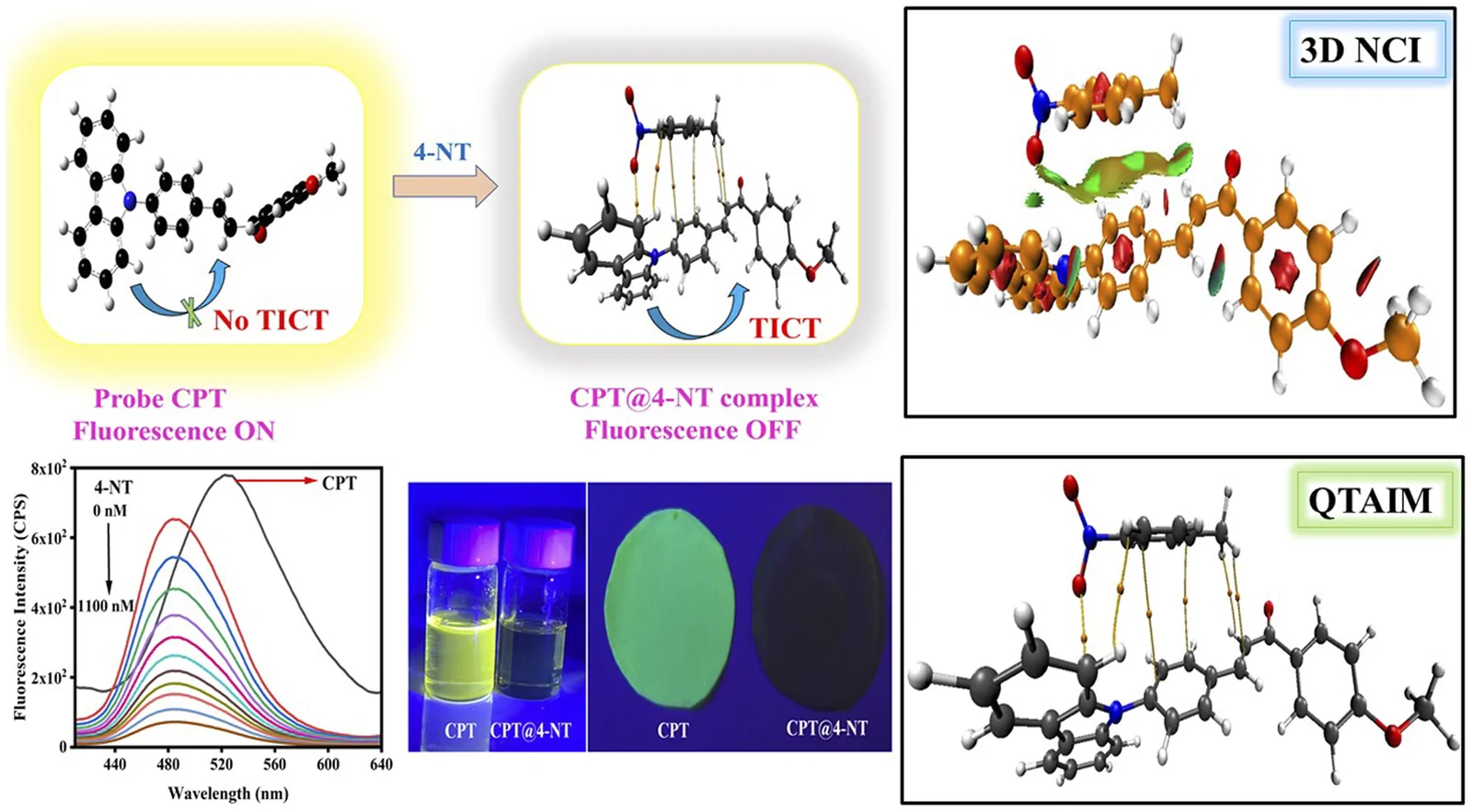

## 1. Introduction

Nitroaromatic compounds (NACs) constitute an important class of environmental pollutants and industrial chemicals extensively utilized in the production of explosives, pesticides, and pharmaceuticals.^1–4^ Among them, 4-nitrotoluene (4-NT) is of particular significance due to its extensive applications as a precursor in the synthesis of energetic materials and fine chemicals. 4-NT serves as an important industrial intermediate for the production of rubber chemicals, azo and sulfur-based dyes, explosive compounds, and various coloring agents for textiles such as cotton, silk, wool, and leather.^5,6^ 4-Nitrotoluene (4-NT) is further employed as a versatile precursor in the manufacturing of plastic foams, paints, optical brightening agents, agrochemicals and pharmaceuticals.^5,7–12^

Due to widespread industrial use, 4-NT released into water and soil generates toxic impacts on the soil and water, leading to either direct or indirect harm to the ecological system.^13,14^ Moreover, the long-term use of 4-NT causes serious health problems, including heart problems, emphysema, dizziness, skin rash, endocrine disturbance and distraction of the central nervous system.^15–20^ Thus, it is crucial to develop effective, sensitive, and selective techniques for 4-NT detection. Conventional analytical techniques, such as gas chromatography,^21^ high-performance liquid chromatography,^22^ mass spectrometry,^23^ X-ray diffraction,^24^ surface-enhanced Raman scattering,^25^ electrochemical methods^26^ and ion mobility spectrometry^27^ have been widely employed for the detection of 4-NT. These techniques generally require sophisticated instrumentation, extensive sample preparation, skilled operation, and cost-effectiveness, but offer limited selectivity and are not well-suited for real-time or site monitoring. In contrast, fluorescence-based sensing techniques have emerged as a powerful and versatile approach owing to their advantages, such as high sensitivity, simplicity, rapid response time, and the capability of real-time analysis. A wide variety of fluorescent probes have been designed for the monitoring of 4-NT, including small organic fluorophores,^28^ conjugated polymers,^29^ metal–organic frameworks,^30^ quantum dots,^31^ and carbon-based nanomaterials.^32^ Despite considerable progress in developing fluorescent probes for 4-NT sensing, many reported systems still suffer from limitations, including relatively high detection thresholds, complex multistep synthesis, and inadequate selectivity, sensitivity, and stability.

Although several organic fluorophores exhibit promising emission characteristics, their practical applicability is often hindered by aggregation-caused quenching (ACQ). This phenomenon becomes more pronounced in concentrated or solid-state samples, where strong intermolecular interactions lead to nonradiative decay. Such behavior typically arises from planar molecular architectures that promote π–π stacking and dipole–dipole interactions, facilitating the formation of excimer or exciplex states that suppress fluorescence.^33^ In contrast, aggregation-induced emission (AIE) offers an effective strategy to overcome these limitations, as introduced by Tang's research groups.^34,35^ AIE-active luminogens are generally weakly emissive in dilute solutions but display significantly enhanced fluorescence in the aggregated or solid state, primarily due to restriction of intramolecular motions that limit non-radiative pathways.^36–38^ The emission characteristics in the aggregated state are further influenced by the mode of molecular packing. For instance, J-aggregates, formed through head-to-tail alignment, often promote efficient radiative transitions and are associated with bathochromic shifts,^39,40^ whereas H-aggregates, arising from face-to-face stacking of chromophores, typically result in hypsochromic shifts and can modulate emission behavior through excitonic interactions.^41,42^

In the present study, we report the design and synthesis of fluorescent probe CPT for the selective sensing of 4-NT. The synthesized probe CPT was characterized using NMR spectroscopy, FTIR spectroscopy, and mass spectrometry (LC-MS). Probe CPT exhibited pronounced solvatochromic behavior, with a significant change in emission wavelength as a function of solvent polarity, providing valuable insight into its excited-state stabilization and photophysical characteristics. In addition, probe CPT demonstrated aggregation-induced emission (AIE), attributed to the restriction of intramolecular rotations and vibrations (RIR) upon aggregation. This was accompanied by the formation of aggregates, leading to enhanced fluorescence intensity along with a hypsochromic shift. These favorable photophysical features enabled probe CPT to act as an efficient platform for the selective and sensitive detection of 4-nitrotoluene (4-NT) in a H_2_O/DMF mixture (9 : 1, v/v). The sensing response was governed by twisted intramolecular charge transfer (TICT) and photoinduced electron transfer (PET) mechanisms, resulting in a distinct change in emission intensity and a detection limit of 0.16 nM. Additionally, probe CPT demonstrates remarkable stability under various environmental conditions, including pH and temperature variations, and maintains stability under prolonged UV exposure. The probe CPT also showed a rapid response time, enhancing its practical applicability. To the best of our knowledge, probe CPT was obtained in good yield and displays remarkable solvatochromic and AIE behavior, and selective detection of the mutagenic analyte 4-NT in both solution and solid phases, demonstrating its practical applicability for real-sample analysis and environmental monitoring.

## 2. Experimental section

### 2.1. Material

All the chemicals and reagents such as 9*H*-carbazol, *p*-methoxy acetophenone, 4-bromobenzaldehyde, *N*,*N*-dimethylformamide (DMF), acetone, dichloromethane (DCM), ethanol, tetrahydrofuran (THF), methanol, chloroform, 2-nitroaniline (2-NA), nitrobenzene (NB), 4-nitrotoluene (4-NT), 4-nitroaniline (4-NA), 1,3-dinitrobenzene (DNB), aniline, nitrobenzoic acid (NBA), trinitrotoluene (TNT), 2,4-dinitrotoluene (DNT), and 4-nitrophenol (4-NP), and distilled water. All reagents were of synthetic grade and purchased from Oakwood Chemicals (USA), Alfa Aesar (UK), Sigma-Aldrich and Daejung Chemicals & Metals (Korea).

### 2.2. Experimental procedure for synthesis of 4-(9*H*-carbazol-9-yl) benzaldehyde (3)

Synthesis of compound 3 was accomplished using literature procedure.^43^ 9*H*-carbazole (1) (2.0 mmol, 334.414 mg), 4-bromobenzaldehyde (2) (2.0 mmol, 370.036 mg), and copper(i) iodide (40 mg) as catalyst are mixed in 10 mL dry DMF. The reaction mixture was stirred, then heated under an inert atmosphere at 140 °C for 48 hours. Upon completion of the reaction, the reaction mixture was cooled to ambient temperature. The inorganic solids produced were filtered off, and the filtrate was put in ice-cold water to allow the product to precipitate and then washed with water and ethanol a few times. Crude product 3 was recrystallized from the solution of dichloromethane (CH_2_Cl_2_) and ethanol, to provide the light-yellow solid in the yield of 95%. ^1^H-NMR (400 MHz, CDCl_3_), *δ* ppm = 10.10 (s, 1H), 8.13 (m, 4H, Ar–H), 7.78 (d, 2H, *J* = 8.3 Hz, Ar–H), 7.49 (d, 2H, *J* = 8.2 Hz, Ar–H), 7.43 (td, 2H, *J* = 7.6 Hz, 1.2, Ar–H), 7.32 (app td, 2H, *J* = 7.9, 1.0 Hz, Ar–H). ^13^C NMR (100 MHz, CDCl_3_) *δ* ppm 190.97 (1 × C), 143.39 (1 × C), 140.04 (2 × C), 134.62 (1 × C), 131.37 (2 × C), 126.82 (2 × C), 126.28 (2 × C), 123.96 (2 × C), 120.81 (2 × C), 120.50 (2 × C), 109.75 (2 × C).

#### 2.2.1. Synthesis of 3-(4-(9*H*-carbazol-9-yl)phenyl)-1-(4-methoxyphenyl)prop-2-en-1-one (CPT)

Mixture of 4-(9*H*-carbazol-9-yl) benzaldehyde 3 (1.0 mmol), 4-methoxyacetophenone 4 (1.0 mmol) and potassium hydroxide (150 mg) was dissolved in 10 mL anhydrous ethanol. The mixture was constantly stirred at 40 °C temperature for 12 hours. The reaction was monitored by thin-layer chromatography (TLC), and the resulting product was recrystallized from *n*-hexane to afford a yellow crystalline product in 91% yield. ^1^H-NMR (400 MHz, CDCl_3_), *δ* ppm: 8.14 (d, 2H, *J* = 7.8 Hz, Ar–H), 8.08 (d, 2H, *J* = 8.8 Hz, Ar–H) 7.91–7.86 (m, 3H, Ar–H), 7.65–7.60 (m, 3H, Ar–H/olefin), 7.48–7.40 (m, 4H, Ar–H/olefin), 7.30 (t, 2H, *J* = 7.6 Hz, Ar–H), 7.00 (d, 2H, *J* = 8.8 Hz), 3.90 (s, 3H, OCH_3_). ^13^C NMR (100 MHz, CDCl_3_) *δ* ppm: 188.46 (1 × C), 163.54 (1 × C), 142.75 (1 × C), 140.43 (2 × C), 139.44 (1 × C), 133.94 (1 × C), 131.02 (1 × C), 130.85 (2 × C), 129.79 (2 × C), 127.12 (2 × C), 126.08 (2 × C), 123.63 (2 × C), 122.35 (1 × C), 120.39 (2 × C), 120.32 (2 × C), 113.91 (2 × C), 109.77 (2 × C), 55.52 (1 × C). HRMS (ESI): *m*/*z* [M + H]^+^ calculated for C_28_H_22_NO_2_, 404.1645; found for C_28_H_22_NO_2_, 404.1664.

### 2.3. Fluorescence sensing experiments of probe CPT

The photoluminescence behavior of probe CPT was investigated in H_2_O/DMF (9 : 1, v/v) solvent system, upon excitation at 360 nm, probe CPT (10 µM) in pure DMF solvent demonstrated maximum fluorescence intensity at 548 nm. To assess its selectivity, a series of nitroaromatic analytes was tested using freshly prepared 1.0 mM solutions, including 2-nitroaniline (2-NA), nitrobenzene (NB), 4-nitrotoluene (4-NT), 4-nitroaniline (4-NA), 1,3-dinitrobenzene (DNB), aniline, nitrobenzoic acid (NBA), trinitrotoluene (TNT), 2,4-dinitrotoluene (DNT), and 4-nitrophenol (4-NP). Emission responses were systematically recorded for each analyte. In addition, interference studies were conducted by monitoring fluorescence behavior in the presence of competing species to assess the probe's CPT selectivity.

## 3. Results and discussion

### 3.1. Chemistry of probe CPT

Probe CPT was synthesized *via* a two-step reaction process involving C–N coupling followed by Claisen–Schmidt condensation. In the first step, carbazole (1) underwent Ullmann-type *N*-arylation with 4-bromobenzaldehyde (2) in the presence of copper(i) iodide (CuI) and K_2_CO_3_ in DMF at 140 °C for 48 hours to afford the key intermediate 3.^43^ This reaction proceeds *via* copper-mediated oxidative addition of the aryl bromide, followed by the coordination and deprotonation of carbazole, and subsequent reductive elimination to form the C–N bond, yielding the *N*-aryl-carbazole aldehyde. In the second step, aldehyde 3 was subjected to base-catalyzed Claisen–Schmidt condensation with 4-methoxyacetophenone 4 in ethanolic KOH at 40 °C temperature for 12 hours.^44^ The reaction proceeds *via* formation of an enolate from the ketone, which subsequently reacts with the aldehyde *via* nucleophilic addition, followed by dehydration to afford conjugated α,β-unsaturated probe CPT in 91% yield (Scheme 1). Probe CPT was characterized through NMR spectroscopy and mass spectrometry. The ^1^H NMR spectrum displayed characteristic signals in the aromatic region *δ* 7.00–8.14 ppm, corresponding to multiple aryl protons, confirming the presence of an extended conjugated framework. The doublets at *δ* 8.14 and 8.08 ppm are attributed to aromatic protons adjacent to the conjugated carbonyl chalcone and alkene moiety, and a distinctive singlet at *δ* 3.90 ppm is assigned to the methoxy (–OCH_3_) group. High resolution mass spectrometry confirms the formation of desired probe CPT. Comprehensive NMR spectra, and mass spectrometry data of probe CPT are provided in SI.

**Scheme 1:**
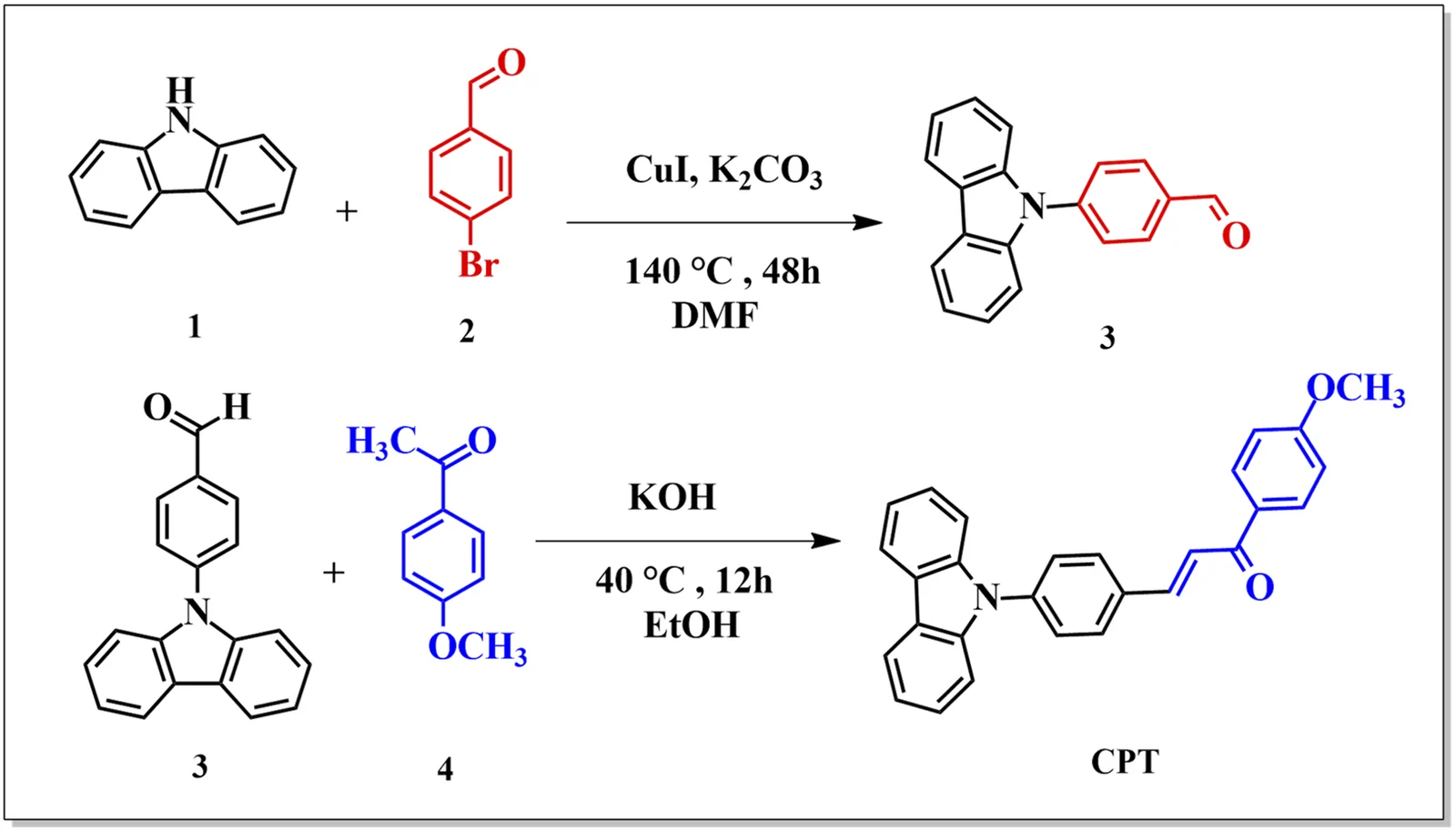
Synthetic route to afford carbazole derived compound of 3-(4-(9*H*-carbazol-9-yl)phenyl)-1-(4-methoxyphenyl)prop-2-en-1-one (CPT).

### 3.2. Solvatochromism of probe CPT

The photophysical properties of probe CPT were investigated in solvents of varying polarity to evaluate its solvatochromic response. Probe CPT exhibits pronounced solvent dependent fluorescence emission, with maxima observed at 501 nm in THF, 505 nm in ethyl acetate (EA), 512 nm in chloroform (CHCl_3_), 522 in acetonitrile (ACN), 524 nm in ethanol, and 548 nm in DMF (Fig. 1a). A gradual bathochromic shift in emission is observed with increasing solvent polarity, reflecting preferential stabilization of the excited state relative to the ground state. Photoexcitation induces electron density transfer from the electron-rich carbazole donor moiety to the electron-deficient chalcone acceptor through the conjugated π-framework. This behavior suggests the presence of intramolecular charge transfer (ICT) within probe CPT.^45^ As solvent polarity increases, enhanced dipole–dipole interactions stabilize the excited ICT state, reducing the energy gap and leading to a bathochromic shift of 47 nm. Moreover, the CIE chromaticity diagram was plotted to illustrate variation in emission wavelength across different solvents (Fig. 1b).

**Fig. 1:**
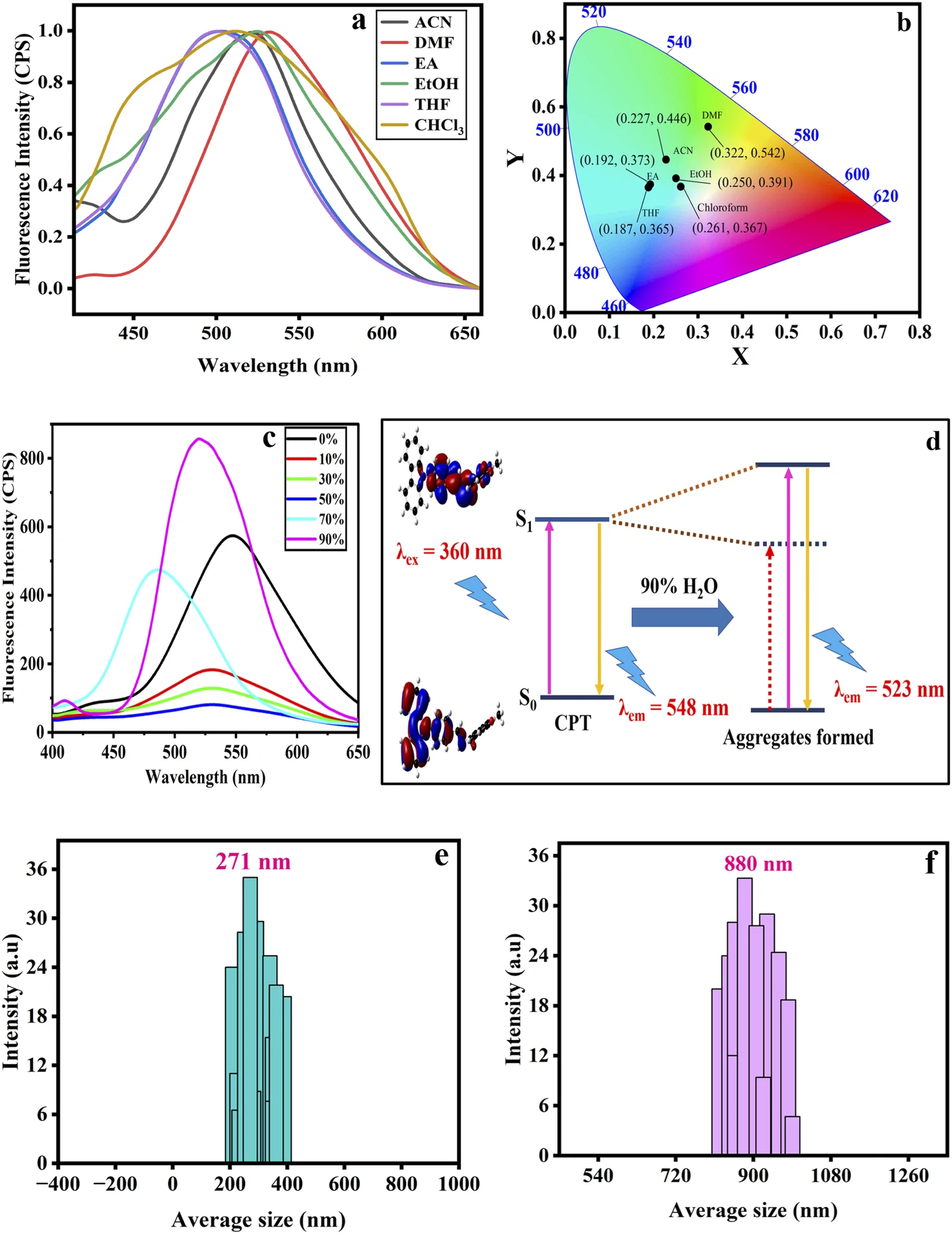
Solvent dependent emission spectra of probe CPT, *λ*_exc_ = 360 nm (a), chromaticity diagram depicting variation in fluorescence response (b), emission response of probe CPT at different water fractions (0–90%) (c), schematic representation of the photophysical mechanism of probe CPT, confirms aggregates formation (d), DLS size distribution analysis of probe CPT with 0% and 90% water fraction (e and f).

### 3.3. Aggregation studies

Probe CPT has demonstrated strong fluorescence emissions in pure DMF, but with the addition of water content to pure DMF, probe CPT exhibits fluctuating behavior in its emission. In pure DMF, it radiates at a relatively long wavelength, however, at 50% water fraction, it shows a hypsochromic shift of lower intensity on the addition of polar protic solvent water, which highly stabilizes the ground state of probe CPT more than the excited state, increasing the energy gap, which results in blue shift and quenching in emission intensity of organic fluorophore by water. At 70% water fraction, emission intensity of probe CPT is enhanced with a slight blue shift due to formation of a poorly soluble aggregate. This leads to a restriction of rotation and rigidification of the structure, thereby promoting aggregation-induced emission. In this conformationally restricted aggregated state, the excited molecules cannot relax *via* intramolecular rotation or other motions; the suppression of relaxation freedom leads to shorter-wavelength emission than that of the free dye. This phenomenon is known as aggregation-induced, blue-shifted emission (AIBSE).^46,47^ Because this blue emission occurs at 480 nm with reduced quenching efficiency upon further addition of water, resulting in high-intensity fluorescence. The fluorescence response was significantly enhanced with further increased in water content up to 90%, accompanied by a significantly large bathochromic shift. At very high-water content, probe CPT forms large aggregates, which result in a lower energy gap between ground and excited states, a bathochromic shift is observed, and fluorescence is very intense due to the strong prohibition of nonradiative decay. Hence, red and bright fluorescence is observed at 523 nm (Fig. 1c and d), quantum yields of probe CPT at different water fractions are provided in Table S1. Additionally, DLS analysis showed a significant increase in particle size from 271 nm to 880 nm as the water fraction increased from 0% to 90% (Fig. 1e and f). Further the SEM analysis were performed to confirmed the aggregation (Fig. S1a and b)

## 4. Sensing studies

Fluorescence titration measurements were systematically performed to elucidate the selective sensing behavior of probe CPT toward 4-nitrotoluene (4-NT). Emission spectra of probe CPT were recorded upon gradual addition of 4-NT over a concentration range of 0–1100 nM. A progressive decrease in fluorescence intensity was observed as the 4-NT concentration increased, indicating efficient quenching. At 1100 nM of 4-NT, maximum quenching was achieved, accompanied by a hypsochromic shift of approximately 39 nm, with the maximum emission shifting from 523 to 484 nm (Fig. 2a). To evaluate a quantitative response, a Stern–Volmer (SV) plot of *I*_0_/*I versus*4-NT concentration was constructed. The plot exhibited good linearity in the concentration range (0–1100 nM), in the lower concentration range of 4-NT (0–600 nM), the SV plot exhibited a more linear relationship suggesting a well-defined quenching mechanism. The probe CPT displayed a high quenching efficiency of ∼91% toward 4-NT (Fig. 2b and S2a). Following IUPAC guidelines, using standard statistical relationships (LOD = 3*σ*/*S* and 10*σ*/*S*), the LOD and LOQ were calculated, where *σ* is the standard deviation of blank responses, and *S* is the slope of the calibration plot. The LOD and LOQ were calculated to be 0.16 nM and 0.55 nM, respectively, indicating the remarkable sensitivity of probe CPT toward 4-NT. To further assess selectivity, interference studies were conducted using various competing analytes. A three-dimensional Stern–Volmer plot (Fig. S2b) revealed a pronounced alteration in fluorescence intensity upon exposure to 4-NT, while other analytes induced negligible responses (Fig. S2c).^48^

**Fig. 2:**
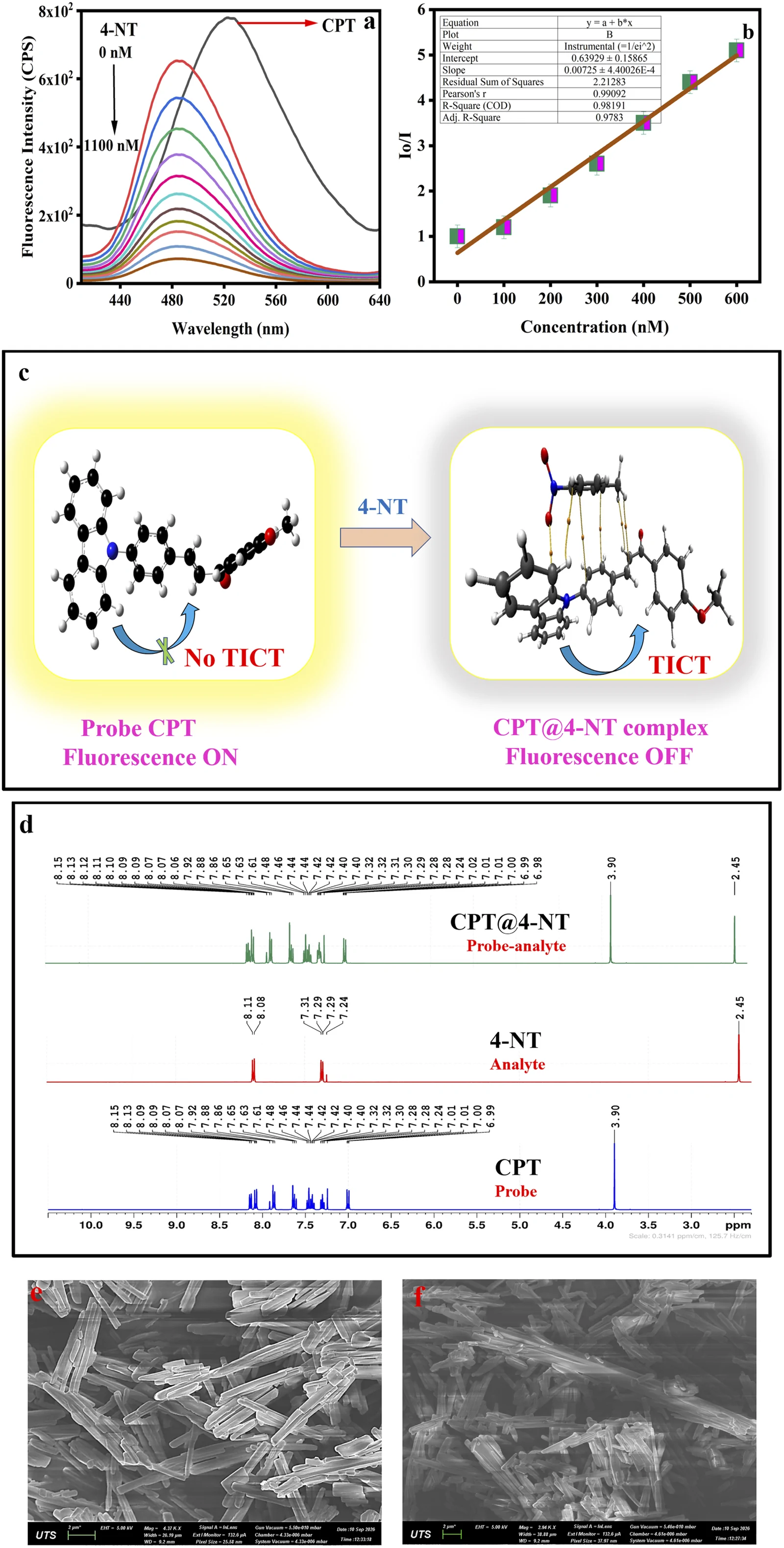
Emission response of probe CPT in the presence of analyte 4-NT (0–1100 nM) (a), 2D SV plot of CPT@4-NT (0–600 nM) (b), proposed graphical illustration of probe CPT in sensing of 4-NT (c), and stacked ^1^H NMR titration (CDCl_3_) spectra of probe CPT, 4-NT, and CPT@4-NT (d), SEM analysis before and after addition of 4-NT (e and f).

### 4.1. Mechanistic insights

In probe CPT, the carbazole segment acts as the electron donor, whereas the α,β-unsaturated carbonyl group functions as the electron acceptor, forming a typical donor–π–acceptor (D–π–A) system. In the S_0_ state, the molecule primarily exists in a relatively planar conformation, allowing effective π-conjugation between the donor and acceptor units.^49^ Upon photoexcitation, electron density is transferred from the carbazole moiety to the α,β-unsaturated carbonyl moiety through the conjugated framework, potentially contributing to the excited state photophysical behavior of probe CPT.^50^ This result in reducing the orbital overlap between the donor and acceptor units, often leading to decreased radiative decay and enhanced non-radiative relaxation pathways. Initially, probe CPT exhibited an emission maximum at 523 nm in 90% water content, indicating the absence of TICT-assisted excited state. Upon sensing of 4-NT, a pronounced hypsochromic shift was observed at 483 nm (Fig. 2c). Alongside a twisted intramolecular charge transfer (TICT) mechanism, photoinduced electron transfer mechanism (PET) also involved as secondary mechanism between interaction of probe CPT and 4-NT. PET-driven electron transfer from probe CPT to 4-NT*via* LUMO–LUMO interaction results in nonradiative decay, thereby quenching the fluorescence intensity. The DFT studies theoretically confirmed that the LUMO of 4-NT is lower in energy than that of probe CPT, thereby permitting electron flow from the electron-dense aromatic part of CPT to the electron-deficient nitro group of 4-NT with an energy difference of −3.46 to −3.29 eV. To gain further insight into the sensing mechanism between probe CPT and 4-NT, ^1^H NMR titration experiments were carried out. Upon addition of 4-NT, new resonances at *δ* 8.12, 8.10, 8.06, and 6.98 ppm appeared upon complex formation CPT@4-NT, while the characteristic 4-NT signal at 8.08 disappeared supporting the probe CPT and analyte's non-covalent interactions (Fig. 2d and S3a). Furthermore, dynamic light scattering (DLS) showed a significant increase in the hydrodynamic diameter upon complex formation, with particle size increasing from 880 nm to 1321 nm. This pronounced increase in particle size indicates the formation of a supramolecular assembly arising from intermolecular interactions between probe CPT and 4-NT (Fig. S2d and e). Moreover, LC-MS analysis of the probe CPT and complex CPT@4-NT were carried out, the *m*/*z* of [CPT + H]^+^ calculated 404.1648, found 404.25, and [CPT + Na]^+^ calculated 426.146 and found 426.25. After interaction with 4-NT, *m*/*z* of complex [CPT + 4-NT + Na]^+^ calculated 563.194 found 563.00 (Fig. S3b and c). Additionally, FTIR analysis was carried out to investigate the interaction of probe CPT with 4-NT. The FTIR spectrum showed a characteristic band at 3058.87 cm^−1^, corresponding to aromatic C–H stretching and a band at 1511.01 cm^−1^ is attributed to C=C stretching vibrations. Additionally, a band observed at 620.37 cm^−1^ is likely to be associated with aromatic C–H bending vibrations. Upon interaction with 4-NT, significant spectral changes were observed; the aromatic C–H stretching band shifted to 3046.58 cm^−1^, indicating weak intermolecular interactions. New bands appeared at 2923.74 cm^−1^ and 2837.75 cm^−1^ assigned to aliphatic C–H stretching vibrations, indicating the involvement of the methyl group of 4-NT in the complex formation. Furthermore, the aromatic C=C stretching band shifted from 1601.09 cm^−1^ to 1592.90 cm^−1^, and the C=N stretching band showed a slight shift from 1511.01 cm^−1^ to 1506.91 cm^−1^, indicating changes in the electronic environment due to intermolecular interaction, possibly *via* π–π stacking or charge transfer interactions (Fig. S3d). These spectral changes confirmed the successful interaction between probe CPT and 4-NT. In addition, scanning electron microscopy (SEM) technique was employed to investigate the morphological characteristics of probe CPT before and after addition of analyte 4-NT (Fig. 2e and f).

### 4.2. Interference studies

The selectivity and practical applicability of the probe CPT towards 4-NT were evaluated through interference, pH, temperature, response time, and photostability studies. Interference studies indicate that various NAC compounds as well as metal ions caused negligible changes in fluorescence intensity of probe CPT, whereas 4-NT induced a pronounced blue shift in fluorescence intensity with quenching response, confirming excellent selectivity and anti-interference capability (Fig. S4a and b). The pH studies showed that the probe CPT maintain stable fluorescence behavior within a 3–11 pH range, indicating its suitability for aqueous and environmental conditions (Fig. S5a). Temperature-dependent studies showed that the probe CPT retains its fluorescence characteristics and sensing efficiency over a temperature range of 10–60 °C, suggesting its potential for practical applications (Fig. S5b). Photostability studies confirmed that the probe CPT does not undergo noticeable photobleaching under continuous irradiation, showing stable fluorescence under the irradiation period investigated (Fig. S6a). Moreover, the probe CPT exhibited a rapid response towards 4-NT, enabling real-time detection (Fig. S6b).

## 5. DFT studies

### 5.1. NCI analysis and RDG

Noncovalent interaction (NCI) analysis is carried out to examine the nature of the interactions between probe CPT and analyte 4-NT. Spectra of RDG that were composed of blue, green and red. These three types of colors represent three forces that are present between surface and analytes. Very strong, attractive forces of hydrogen bonding (or any other attractive force) are denoted in blue. Green is associated with weak forces, and red is obstruction between moving species by their steric effect. In analyzing the 3D plots the interaction is proportional to the intensity of the iso surface; the greater the intensity, the larger the number of interacting forces we possess. In 3D iso surface, green patches can be seen between probe CPT and 4-NT. This confirms that there are present weak forces between surface and analyte, with a mixture of red and green patches also visible in various regions of probe CPT, indicating steric repulsion and van der Waals attraction, respectively. The attractive forces between the rings of CPT and the 4-NT surface are shown as green patches. These patches are very adequate, indicating a strong indication of presence of π–π stacking interaction between probe CPT and 4-NT (Fig. 3a). The 2D NCI plot showed three different colors green, red and blue, representing weak van der Waals force, repulsive force and strong hydrogen bond respectively. The range between 0.00 a.u. and −0.02 a.u. indicates that there is a strong van der Waals attraction, and the positive forces, such as the electrostatic interaction and hydrogen bonding, are represented by more negative values on the *x*-axis (Fig. 3b).

**Fig. 3:**
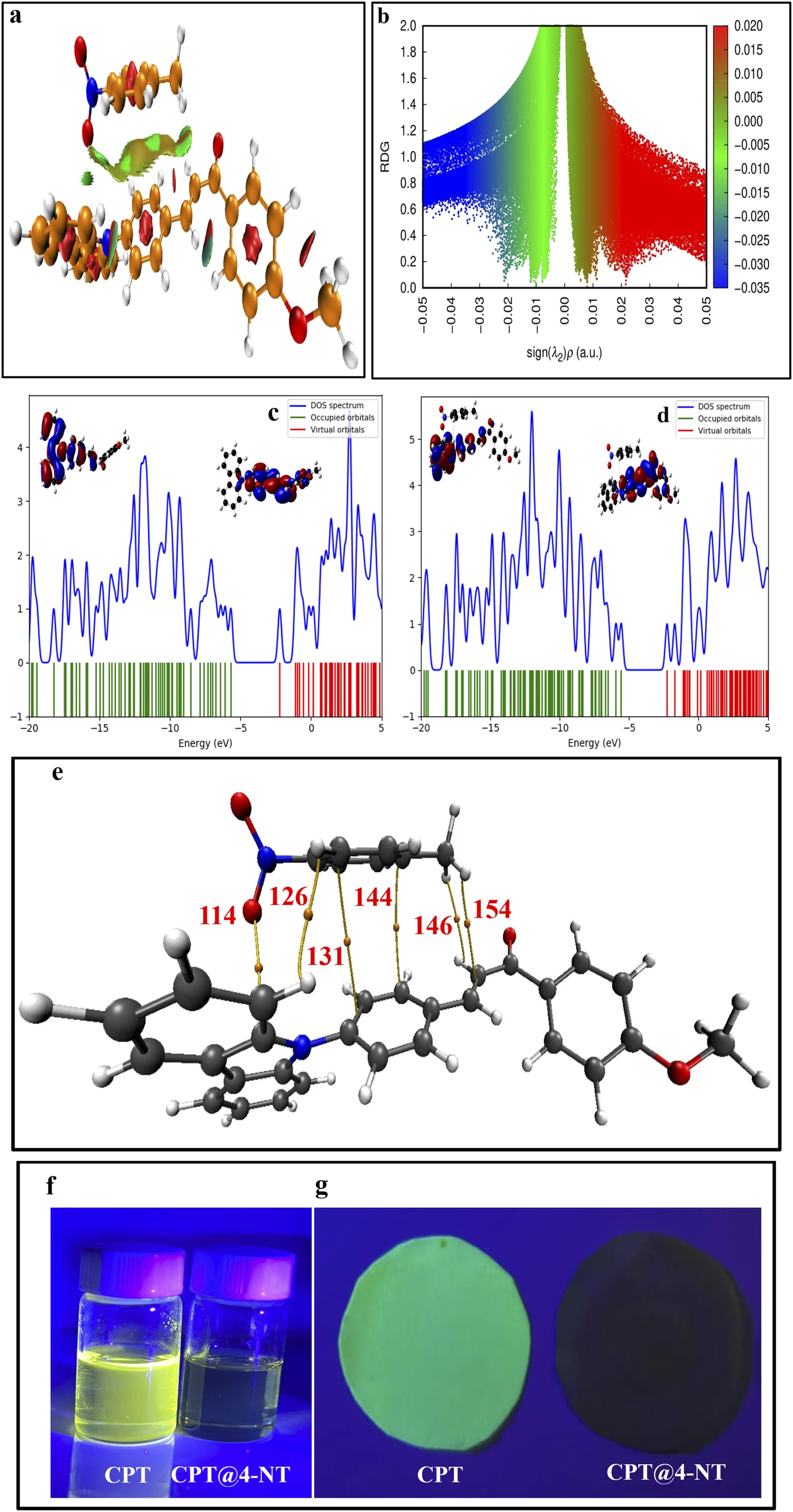
3D NCI of CPT@4-NT (a), 2D NCI of CPT@4-NT (b), DOS spectra of probe CPT and CPT@4-NT (c and d), QTAIM analysis of CPT@4-NT (e), solution and solid phase sensing of 4-NT by probe CPT under 365 nm UV lamp (f and g).

### 5.2. FMO and DOS

Probe CPT is distributed electronically in the ground state frontier molecular orbitals. It is reported that the electronic density is concentrated on the carbazole in the ground state, the orbital HOMO, where it is mainly the donor fraction (D) and on the α,β-unsaturated ketone in the excited state S_1_, where the electron density is located predominantly on the acceptor (A), which supports an ICT. The molecular orbital energy levels, most notably HOMO and LUMO are important parameters of the electronic nature of a molecule, as well as including the chemical behavior of a molecule. Thus, it is most important to study the HOMO and LUMO energies to understand the electronic properties of any structure. The calculated energy gap for the free probe CPT and the complex with 4-NT, as shown in the figure, has decreased compared with the free probe (3.46 eV in CPT) and is now 3.29 eV. In CPT@4-NT, which is strong evidence of significant electronic interaction between probe CPT and 4-NT, frequently due to charge transfer. To further elucidate the electronic behavior between probe CPT and 4-NT, density of states (DOS) calculations were employed. In free state, probe CPT exhibited energy levels at −5.68 eV and −2.22 eV, which were shifted to −5.56 eV and −2.27 eV upon interaction with 4-NT. This change in electronic distribution, accompanied by a reduced HOMO–LUMO energy gap, signifies strong electronic perturbation and effective probe-analyte interactions (Fig. 3c and d).

### 5.3. QTAIM

QTAIM analysis showed that the non-covalent interactions by studying the topology of the electron density, rho (*r*), at given locations in three-dimensional space. Careful consideration of bond critical points (BCPs) is in the range between interacting atoms and gives initial parameters to define the kind of interaction (Fig. 3e). These are: the electron density, rho (*r*), which contains information about bond strength; the Laplacian, 72 rho (*r*), which includes information about whether charge is depleted (have a positive value, indicating a closed-shell interaction) or concentrated (have a negative value, indicating a covalent character); and the total electron energy density, *H* = *V*(*r*) + *G*(*r*), the sum of potential and kinetic energy. A large electron density of the bond critical points (BCPs), *ρ*(*r*) = 0.0088, 0.3029, 0.0036, 0.0059, 0.0057 and 0.0062 a.u. This more positive interaction between probe CPT and 4-NT with a different physical origin is directly supported by the QTAIM data (Table S2).

## 6. Applications

### 6.1. Solid and solution phase sensing

The practical applicability of probe CPT was evaluated through solid-phase sensing investigations. The probe CPT was added to the filter paper, and its fluorescence response towards 4-NT was examined under UV illumination. Upon exposure to 4-NT, the probe CPT exhibited a rapid and visually distinguishable change in fluorescence intensity by quenching process showed good sensitivity, rapid response time, and reproducibility (Fig. 3f). Moreover, the sensing performance of 10 µM probe CPT was evaluated in solution using UV lamp, upon addition of 1100 nM addition of 4-NT, a pronounced fluorescence quenching was observed, accompanied by a visible color change from bright emissive state to dark emissive state under UV-lamp, highlight the potential applicability of probe CPT for sensitive and selective detection of 4-NT in environmental and analytical systems (Fig. 3g & Video S1).

### 6.2. Real sample analysis

To validate the real-world applicability, probe CPT was employed for the analysis of 4-NT in both tap water and industrial effluent samples. The collected samples were subjected to filtration prior to analysis. The pretreated samples were then spiked with known concentration of 4-NT, upon addition of probe CPT, a gradual decrease in fluorescence intensity was observed in both tap water and industrial samples, indicating efficient quenching behavior of probe CPT in the presence of 4-NT (Fig. S7). To assess the accuracy, recovery studies were performed, the calculated recovery percentages were found with low relative standard deviation (RSD), confirming high precision and reliability (Table S3). Overall, these results demonstrate that probe CPT is capable of sensitive and accurate detection of 4-NT in both tap water and industrial effluent samples.

## 7. Conclusion

The synthesis of probe CPT was successfully achieved with 91% yield. Probe CPT exhibited a maximum emission at 548 nm in pure DMF solvent. Upon gradual addition of water from 0% to 90%, a pronounced hypsochromic shift to 523 nm was observed, indicating aggregate formation. Owing to its remarkable fluorescence characteristics, the probe CPT demonstrated excellent performance for the selective detection of 4-NT, achieving a low LOD of 0.16 nM. The involvement of non-covalent interactions in the sensing process was confirmed by ^1^H NMR and FTIR titration studies. The selectivity of probe CPT toward 4-NT was evaluated in the presence of interferents, under varying pH and temperature conditions. The probe CPT demonstrated excellent photostability and fast response characteristics, indicating its robustness for sensing applications. A reduction in the HOMO–LUMO energy gap, along with new virtual orbital formation after interaction with 4-NT, reflects strong sensitivity toward 4-NT. NCI and QTAIM analyses further confirmed the presence of significant intermolecular interactions, with BCP parameters supporting the presence of non-covalent forces. Additionally, probe CPT showed efficient sensing performance in both solid and liquid phases, suggesting its applicability for real industrial effluent analysis.

## Author contributions

Saleh S. Alarfaji: methodology, visualization, software, formal analysis. Alam Shabbir: methodology, investigation, formal analysis, writing – original draft. Huda Khalid: methodology. Qudsia Sajid: methodology, writing – original draft. Asad Muhammad Khan: methodology, investigation. Umar Farooq: investigation, supervision. Sohail Anjum Shahzad: conceptualization, methodology, investigation, validation, supervision, project administration, writing – review & editing.

## Conflicts of interest

The authors declare that they have no known competing financial interests or personal relationships that could have appeared to influence the work reported in this paper.

## Supplementary Material

RA-OLF-D6RA08681K-s001

## Data Availability

The supporting data has been provided as a part of the supplementary information (SI). Supplementary information: theoretical studies, SEM images, DLS, NMR, and LC-MS titrations, photophysical studies *e.g.*: SV plots, pH, temperature effects, photostability, and selectivity studies in figures and tabular forms. See DOI: https://doi.org/10.1039/d6ra08681k.
