# Supplementary material for: Development of AIE active carbazole based fluorescent probe for rapid detection of 4-nitrotoluene in solution and solid phases: experimental and DFT insights
Source: RSC Adv. 2026 Nov 4. Online ahead of print. doi: 10.1039/d6ra08681k (PMC13733534; doi:10.1039/d6ra08681k)
Supplement: Supplementary file 1 [file RA-OLF-D6RA08681K-s001.pdf]

Supplementary Information (SI) for RSC Advances.
This journal is © The Royal Society of Chemistry

Supporting Information

Development of AIE Active Carbazole Based Fluorescent Probe for Rapid Detection of 4-Nitrotoluene in Solution and Solid Phases: Experimental and DFT Insights

Saleh S. Alarfaji[a], Alam Shabbir[b,c,1], Huda Khalid[b,1], Qudsia Sajid[b,1], Asad Muhammad Khan[b], Umar Farooq[b], Sohail Anjum Shahzad[b,*]

[a] *Department of Chemistry, Faculty of Science, King Khalid University, P.O. Box 960, Abha, 61421, Saudi Arabia*

[b] *Department of Chemistry, COMSATS University Islamabad, Abbottabad Campus, University Road, Abbottabad 22060, Pakistan*

[c] *School of Mathematical and Physical Sciences, Faculty of Science, University of Technology Sydney, Sydney, New South Wales, Australia*

* Corresponding author: Sohail Anjum Shahzad, Department of Chemistry, COMSATS University Islamabad, Abbottabad Campus, University Road, Abbottabad 22060, Pakistan.

E-mail address: sashahzad@cuiatd.edu.pk (S. A. Shahzad)

SI-1 Instruments and reagents……………………………………………………………3

SI-2 Theoretical studies…………………………………………………………………3

Figure S1: SEM analysis of probe CPT before and after addition of water (a and b) 4
Figure S2: 2D Stern-Volmer plot of CPT@4-NT complex (0-1100 nM) (a), 3D SV plot of probe **CPT** in the presence of different competing analytes (b), emission spectra of probe CPT with different nitroaromatic compounds (c) (Excitation wavelength = 360 nm, Concentration of probe **CPT** 10 μM in 1:9, v/v of DMF/H_2O), DLS analysis of probe CPT before and after addition of analyte 4-NT (d and e). 6
Figure S3: Stacked HNMR titration spectra of CPT@4-NT (a), LC-MS spectra of probe **CPT** (b), LC-MS titration spectra of CPT@4-NT (c), and FTIR titration spectra of CPT@4-NT (d).......... 8
Figure S4: Emission spectra of probe **CPT** and CPT@4-NT in the presence of cations and anions (a and b) 8
Figure S5: Effect of pH (a) and temperature (b) on quenching response of probe **CPT** toward 4-NT. 9
Figure S6: Photostability test of probe **CPT** towards 4-NT (a) and relative fluorescence quenching of probe **CPT** upon exposure to 4-NT within the time interval of 20-80 s (b)............................... 9
Figure S7: Emission response of probe **CPT** against different concentration of industrial sample 10

Table S1: The quantum yield of probe CPT with different water fractions 10
Table S2: Representation of BCPs of CPT@4-NT 10
Table S3: Recovery experiment for detection of 4-NT in an industrial effluent and real water...11

SI-1 Instruments and reagents

Characterization of probe **CPT** was carried out using ^{1}H and ^{13}C NMR spectroscopy on a Bruker Avance III spectrometer with $CDCl_3$ as deuterated solvent. NMR titration studies were also conducted in $CDCl_3$. Fluorescence emission measurements were carried out on a Perkin Elmer LS-45 spectrofluorometer (Hitachi, Japan). For fluorescence studies targeting analyte detection, probe **CPT** solutions were prepared in HPLC-grade DMF and distilled water. All reagents and chemicals employed in this research were purchased from Sigma Aldrich (USA), Daejung Chemicals & Metals (Korea), Alfa Aesar (UK), and Oakwood Chemicals (USA), were of analytical grade. Solvents and analytes, including DMF, dichloromethane, methanol, ethyl acetate, n-hexane, 2-nitroaniline (2-NA), nitrobenzene (NB), 4-nitrotoluene (4-NT), 4-nitroaniline (4-NA), dinitrobenzene (DNB), aniline, nitrobenzoic acid (NBA), trinitrotoluene (TNT), 2,4-dinitrotoluene (DNT), and 4-nitrophenol (4-NP), and distilled water, were employed. Solvent purification was carried out by distillation.

SI-2 Theoretical studies

Computational analyses were carried out within the framework of density functional theory (DFT), a widely recognized method for examining electronic structures and intermolecular interactions in organic systems [1]. For the exchange-correlation treatment, the B3LYP / 6-311G (d,p) functional-combining Becke's three-parameter exchange with the Lee-Yang-Parr correlation-was employed to investigate the electronic properties and non-covalent interactions [2, 3]. To ensure the reliability of the computed interaction energies, the basis set superposition error (BSSE) was evaluated and subsequently corrected using the counterpoise method, thereby minimizing the artificial stabilization arising from basis set overlap [4]. The electronic properties, including the energy of individual molecular orbitals, were evaluated through frontier molecular orbital (FMO) and density of states (DOS) analyses using Gaussian 09 and GaussSum software [5, 6]. To gain a deeper understanding of the interaction between probe **CPT** and 4-NT, non-covalent interaction (NCI) analysis and quantum theory of atoms in molecules (QTAIM) analyses were performed using the Multiwfn program, with Visual Molecular Dynamics (VMD) employed for visualization of the generated data [7-10]. These analyses provided a clear understanding of the specific Non-covalent interactions, including hydrogen bonding, π-π stacking, and van der Waals forces, governed the strong affinity and highly selective recognition of 4-NT by the probe **CPT**.

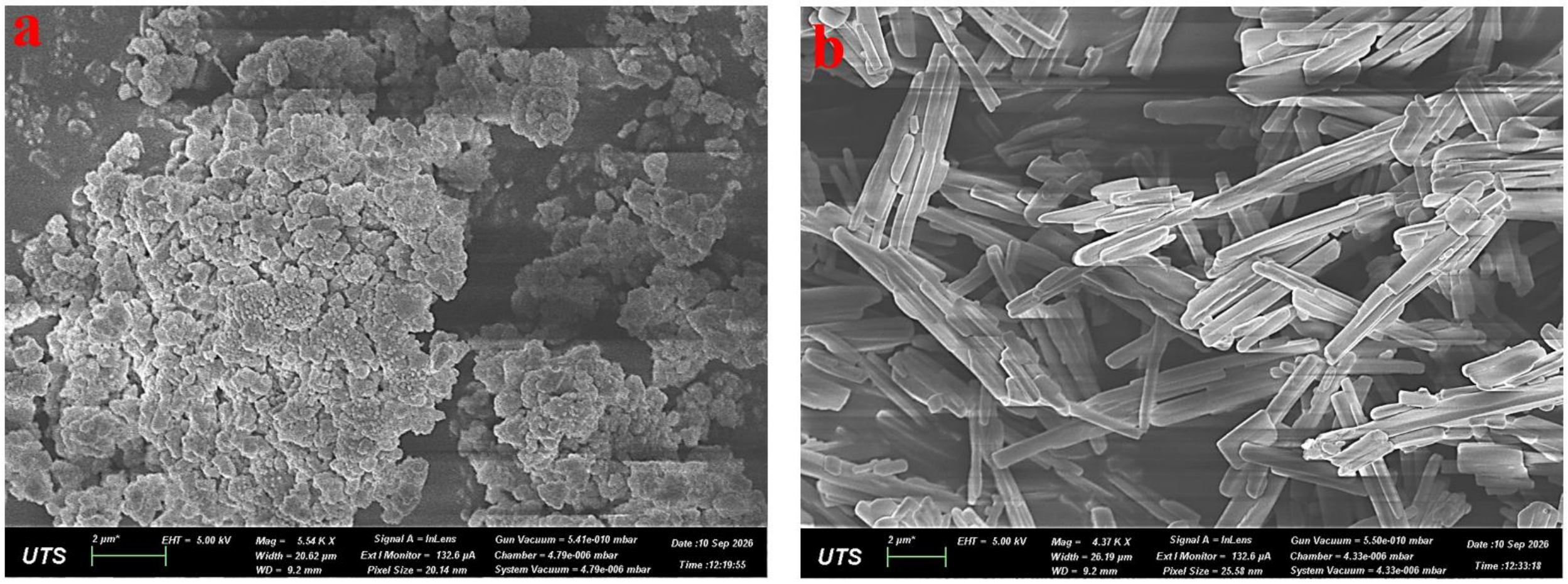

Figure S1: SEM analysis of probe CPT before and after addition of water (a and b)

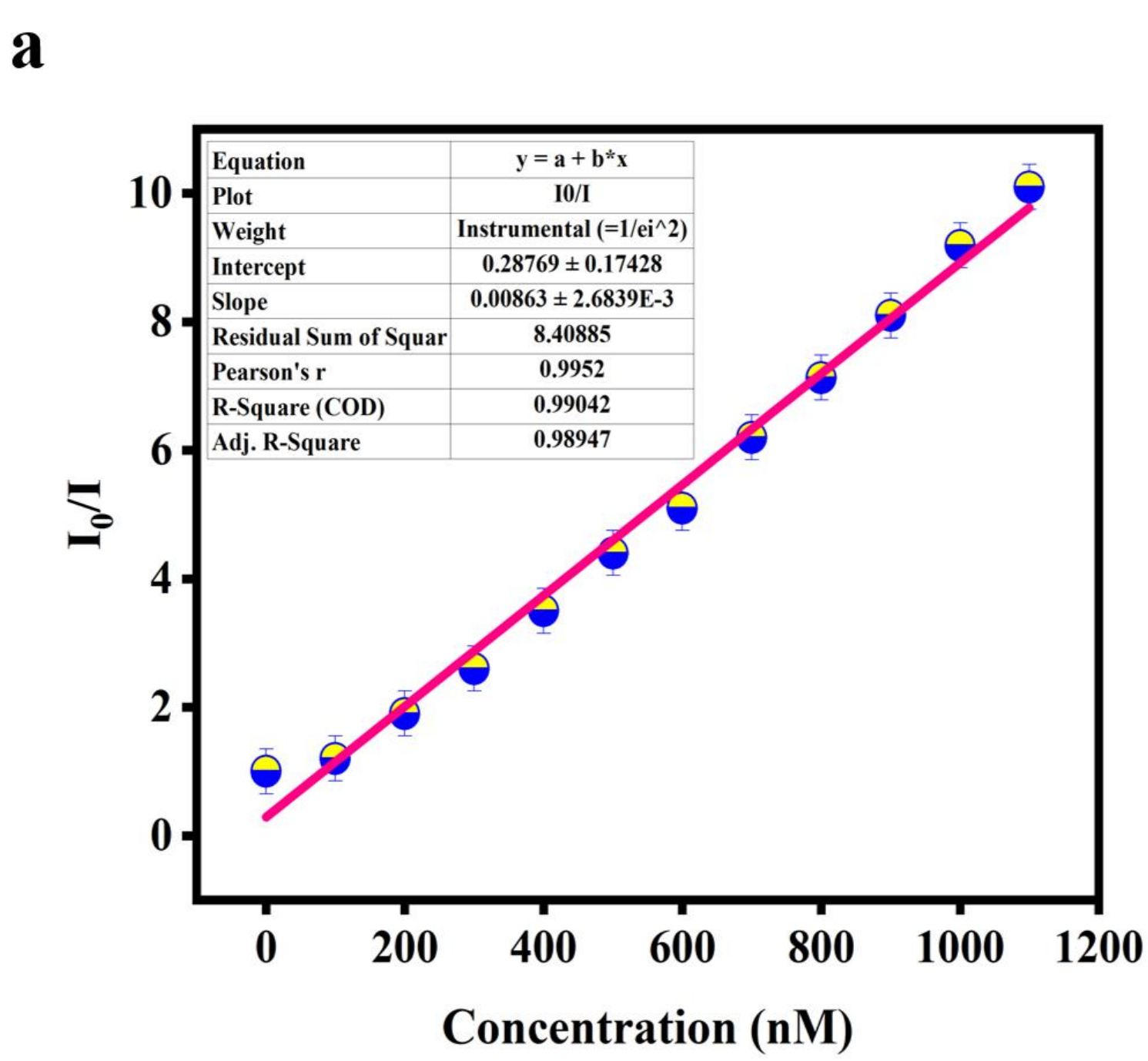

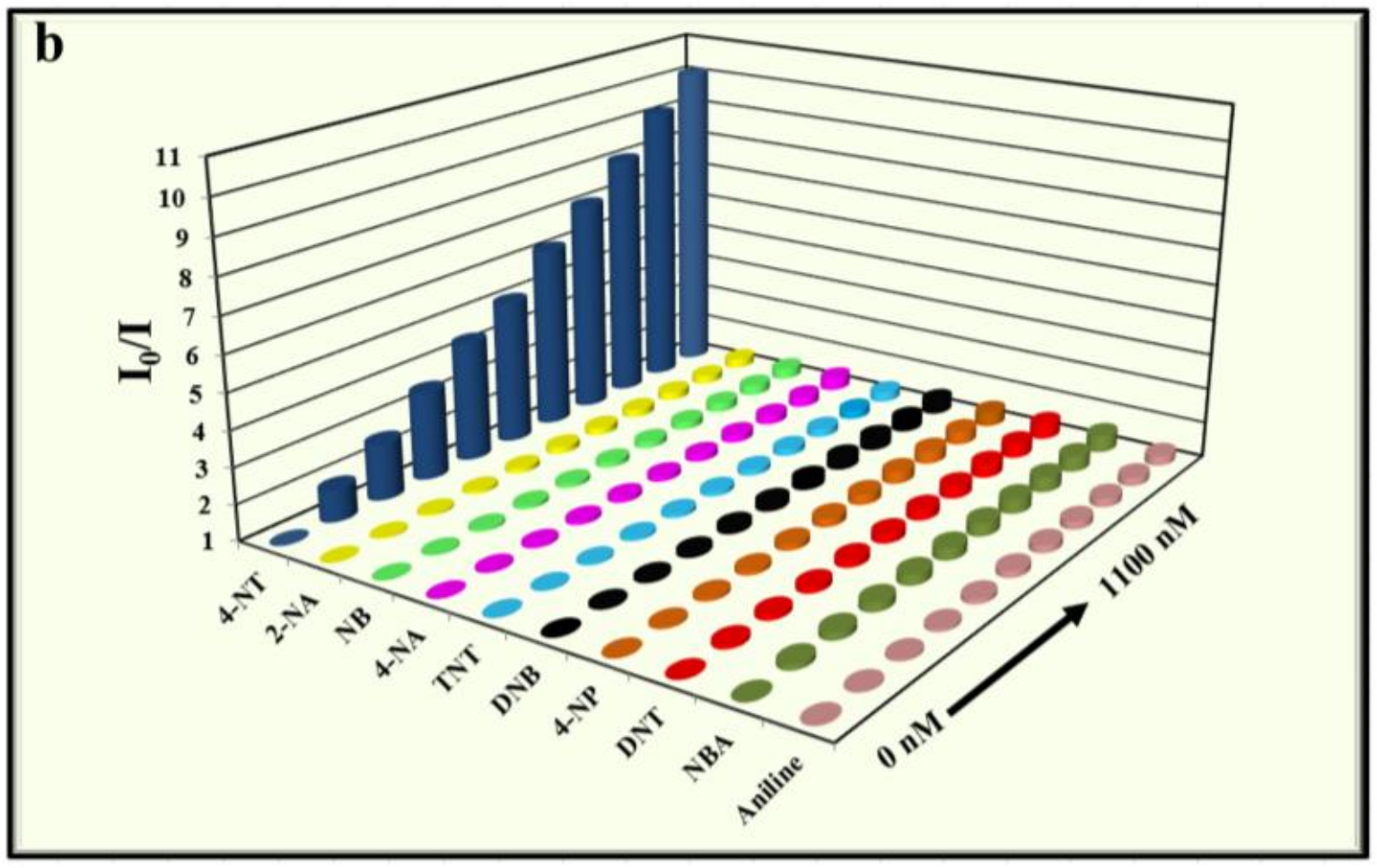
b
I_0/I
1
2
3
4
5
6
7
8
9
10
11
4-NT
2-NA
NB
4-NA
TNT
DNB
4-NP
DNT
NBA
Aniline
0 nM
1100 nM

c

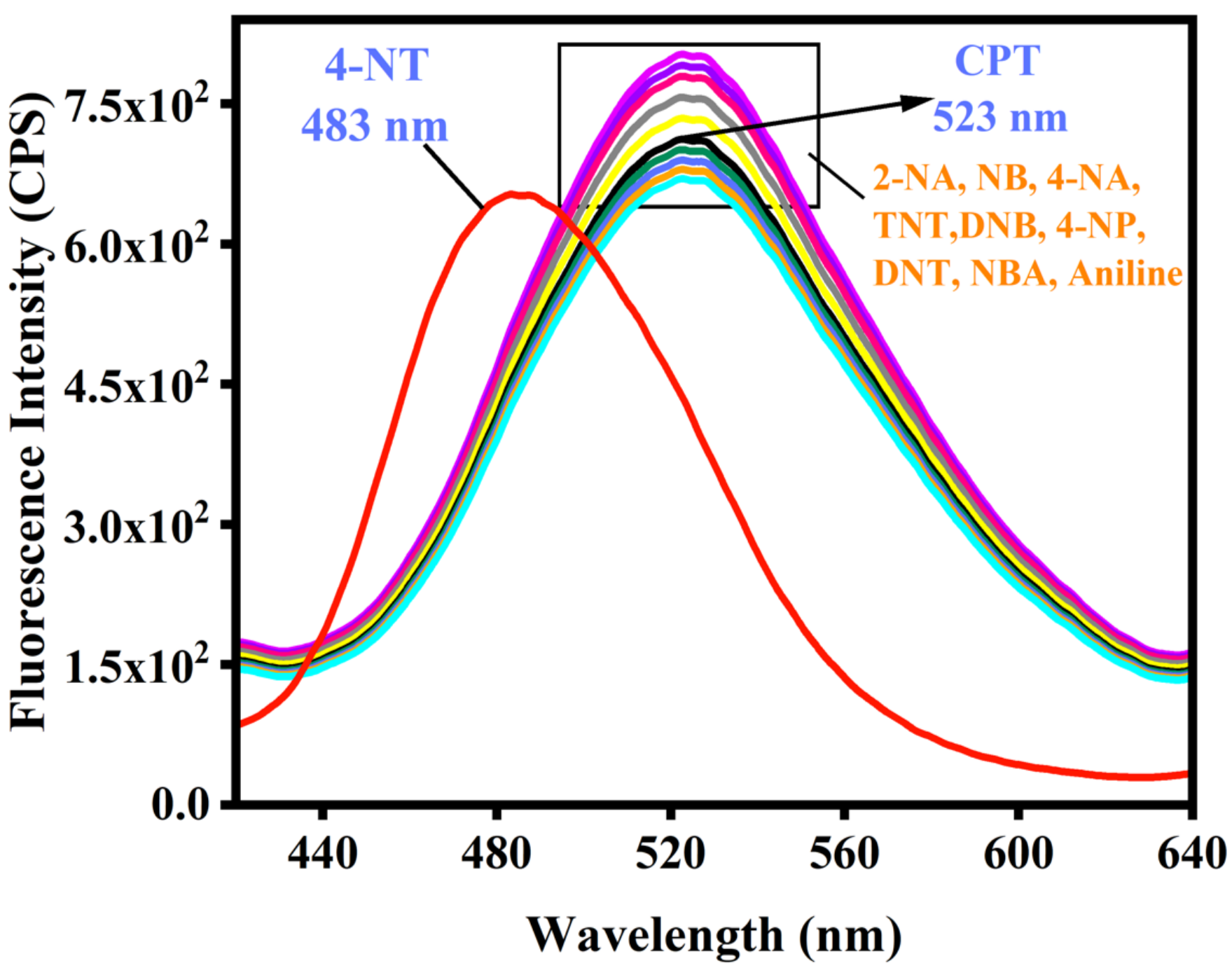
4-NT
483 nm
CPT
523 nm
2-NA, NB, 4-NA, TNT,DNB, 4-NP, DNT, NBA, Aniline
Fluorescence Intensity (CPS)
0.0
1.5x10²
3.0x10²
4.5x10²
6.0x10²
7.5x10²
440
480
520
560
600
640
Wavelength (nm)

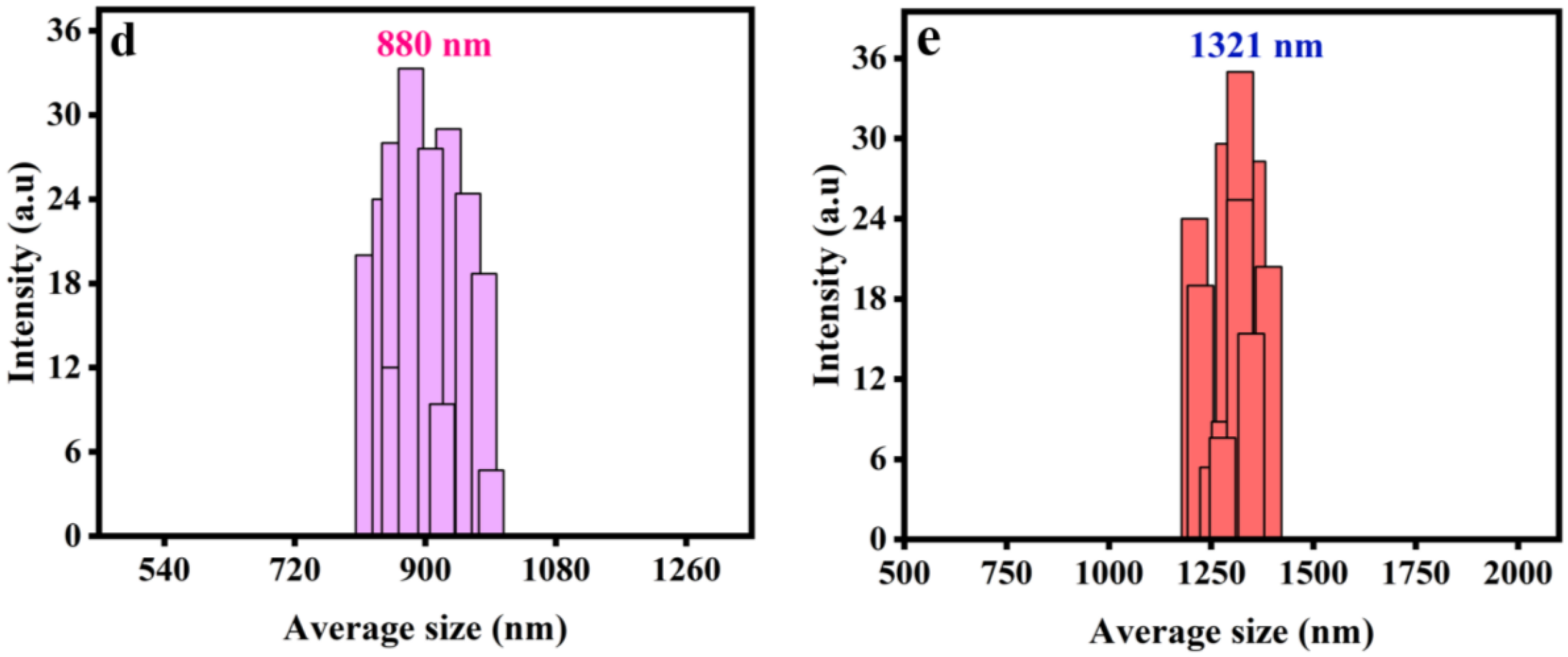

Figure S2: 2D Stern-Volmer plot of CPT@4-NT complex (0-1100 nM) (a), 3D SV plot of probe **CPT** in the presence of different competing analytes (b), emission spectra of probe CPT with different nitroaromatic compounds (c) (Excitation wavelength = 360 nm, Concentration of probe **CPT** 10 µM in 1:9, v/v of DMF/H_2O), DLS analysis of probe CPT before and after addition of analyte 4-NT (d and e).

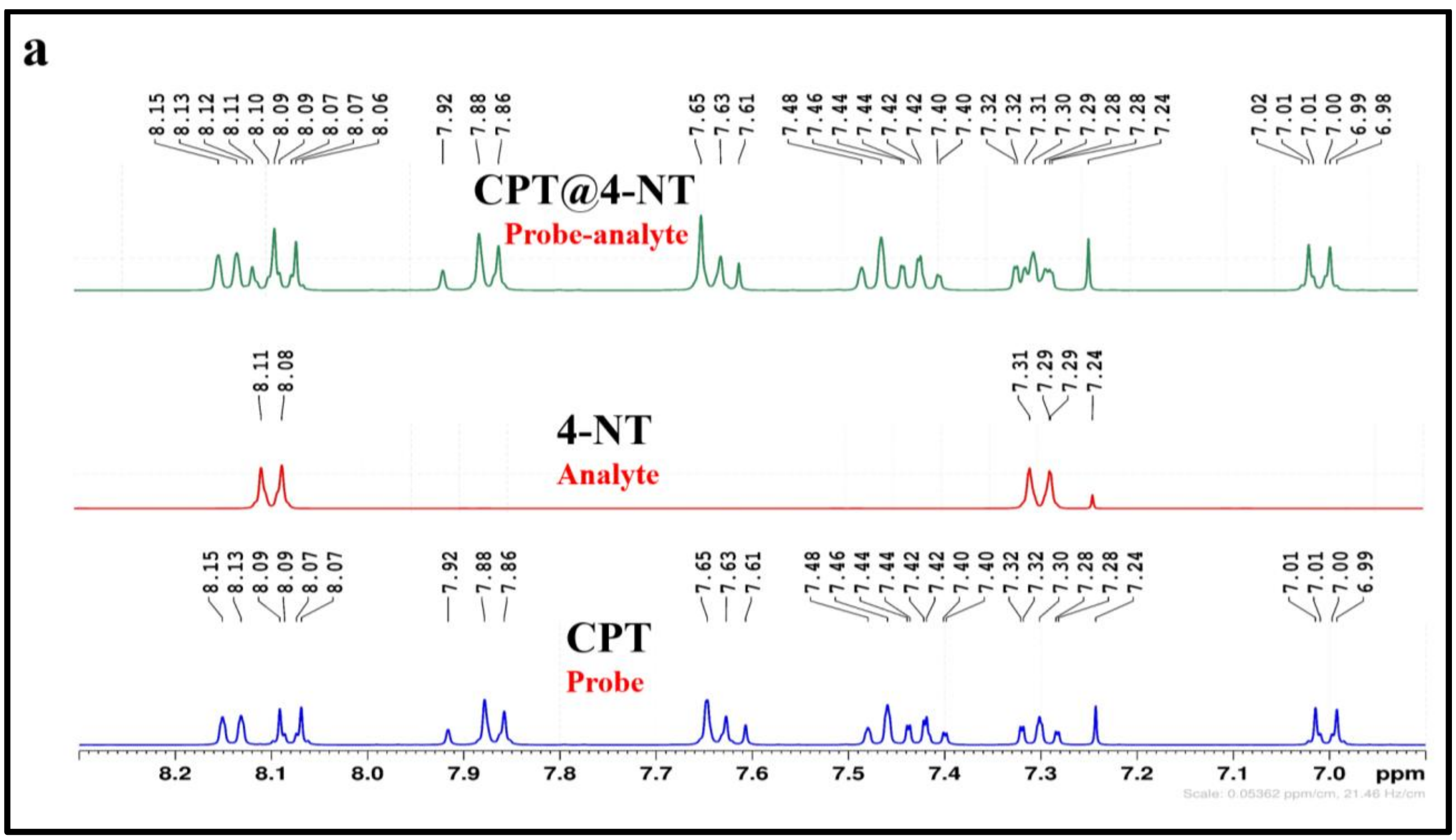

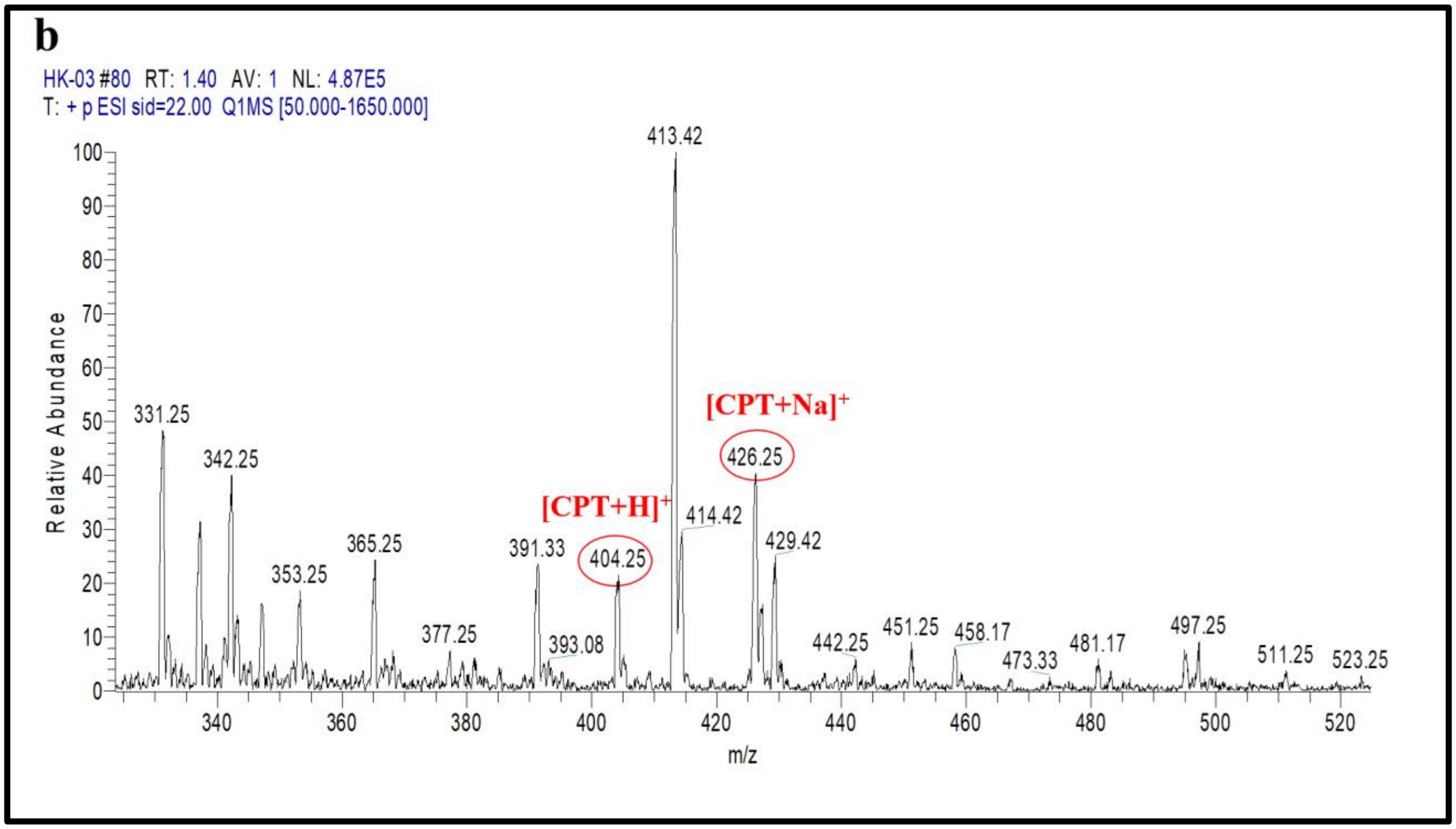
b
HK-03 #80 RT: 1.40 AV: 1 NL: 4.87E5
T: + p ESI sid=22.00 Q1MS [50.000-1650.000]
Relative Abundance
m/z
413.42
331.25
342.25
353.25
365.25
377.25
391.33
393.08
[CPT+H]+
404.25
414.42
[CPT+Na]+
426.25
429.42
442.25
451.25
458.17
473.33
481.17
497.25
511.25
523.25

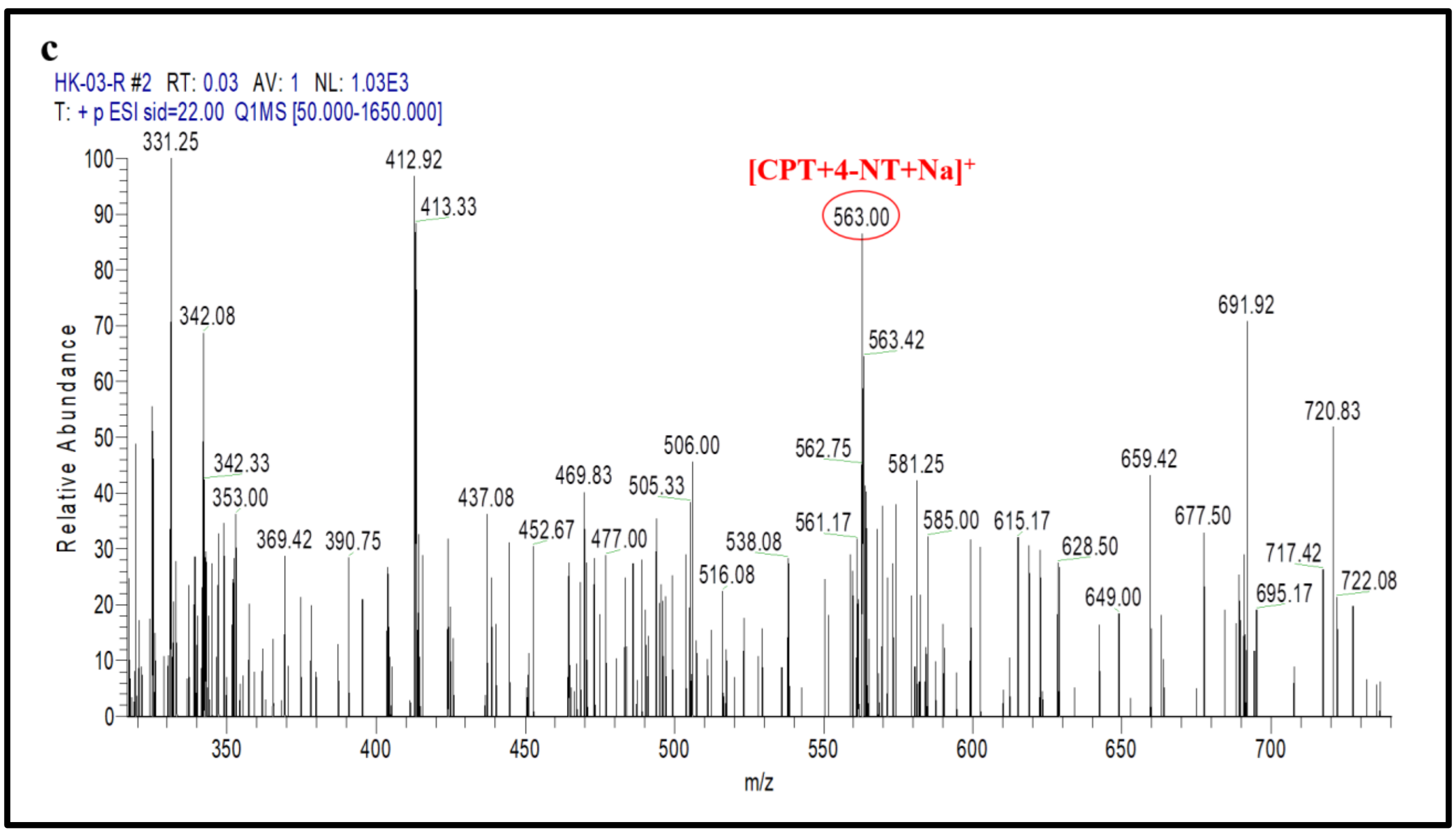
c
HK-03-R #2 RT: 0.03 AV: 1 NL: 1.03E3
T: + p ESI sid=22.00 Q1MS [50.000-1650.000]
Relative Abundance
m/z
331.25
342.08
342.33
353.00
369.42
390.75
412.92
413.33
437.08
452.67
469.83
477.00
505.33
506.00
516.08
538.08
[CPT+4-NT+Na]+
563.00
563.42
562.75
561.17
581.25
585.00
615.17
628.50
649.00
659.42
677.50
691.92
695.17
717.42
720.83
722.08

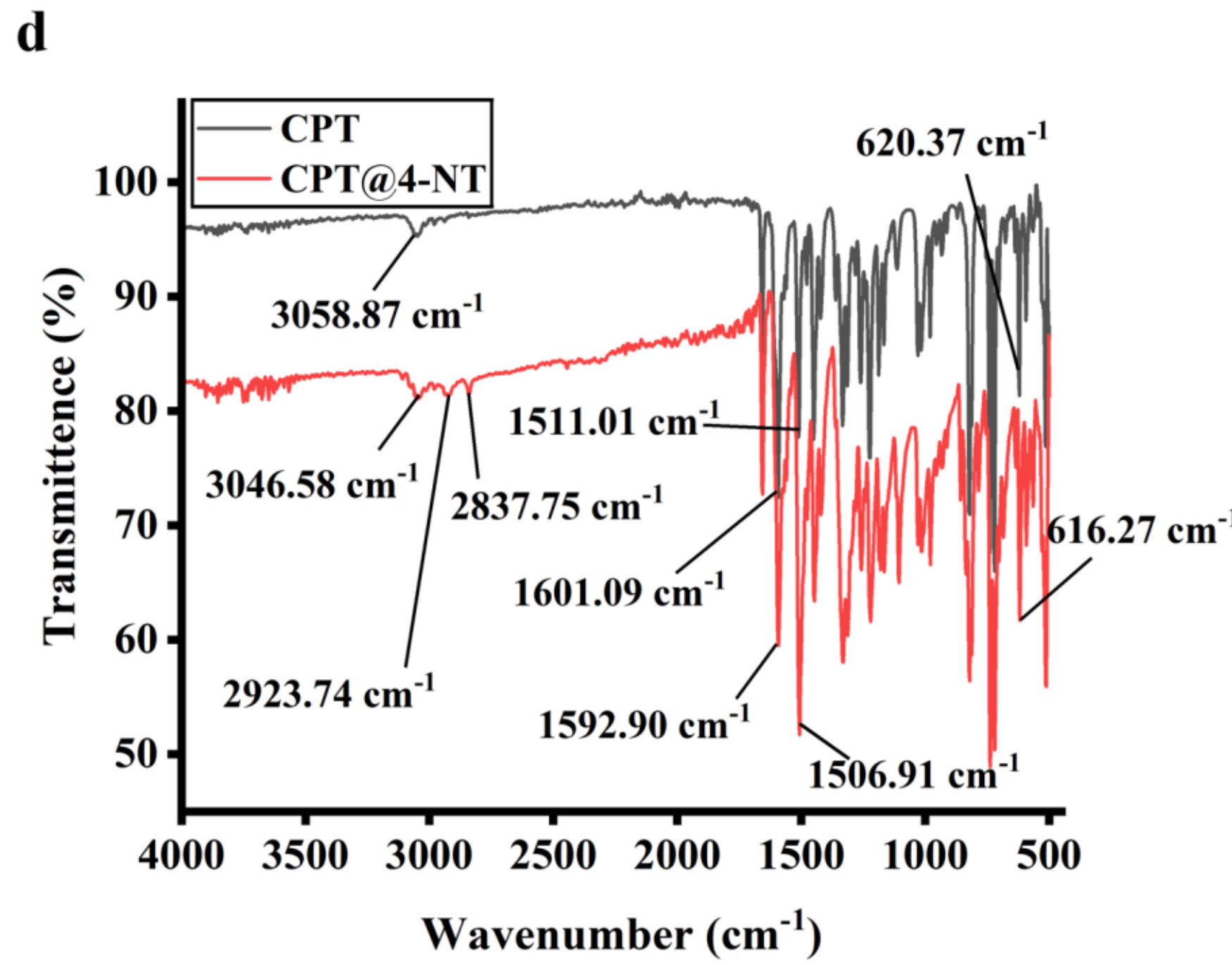

Figure S3: Stacked HNMR titration spectra of CPT@4-NT (a), LC-MS spectra of probe **CPT** (b), LC-MS titration spectra of CPT@4-NT (c), and FTIR titration spectra of CPT@4-NT (d)

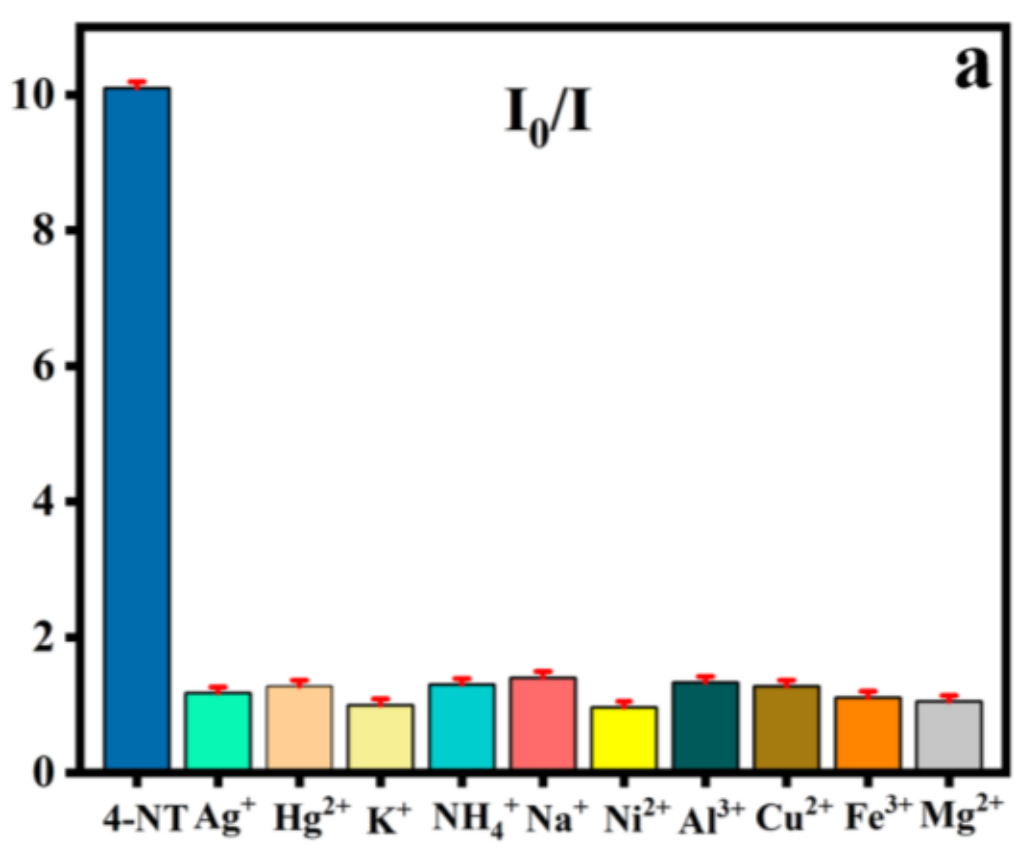

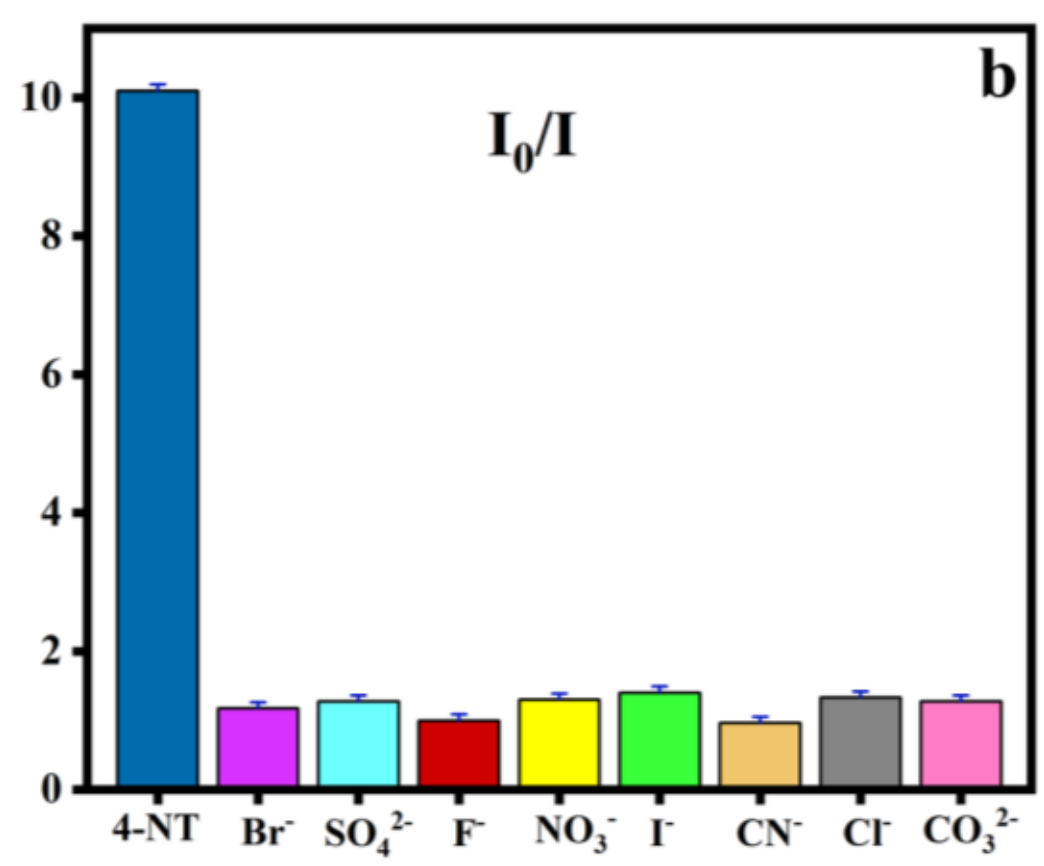

Figure S4: Emission spectra of probe **CPT** and CPT@4-NT in the presence of cations and anions (a and b)

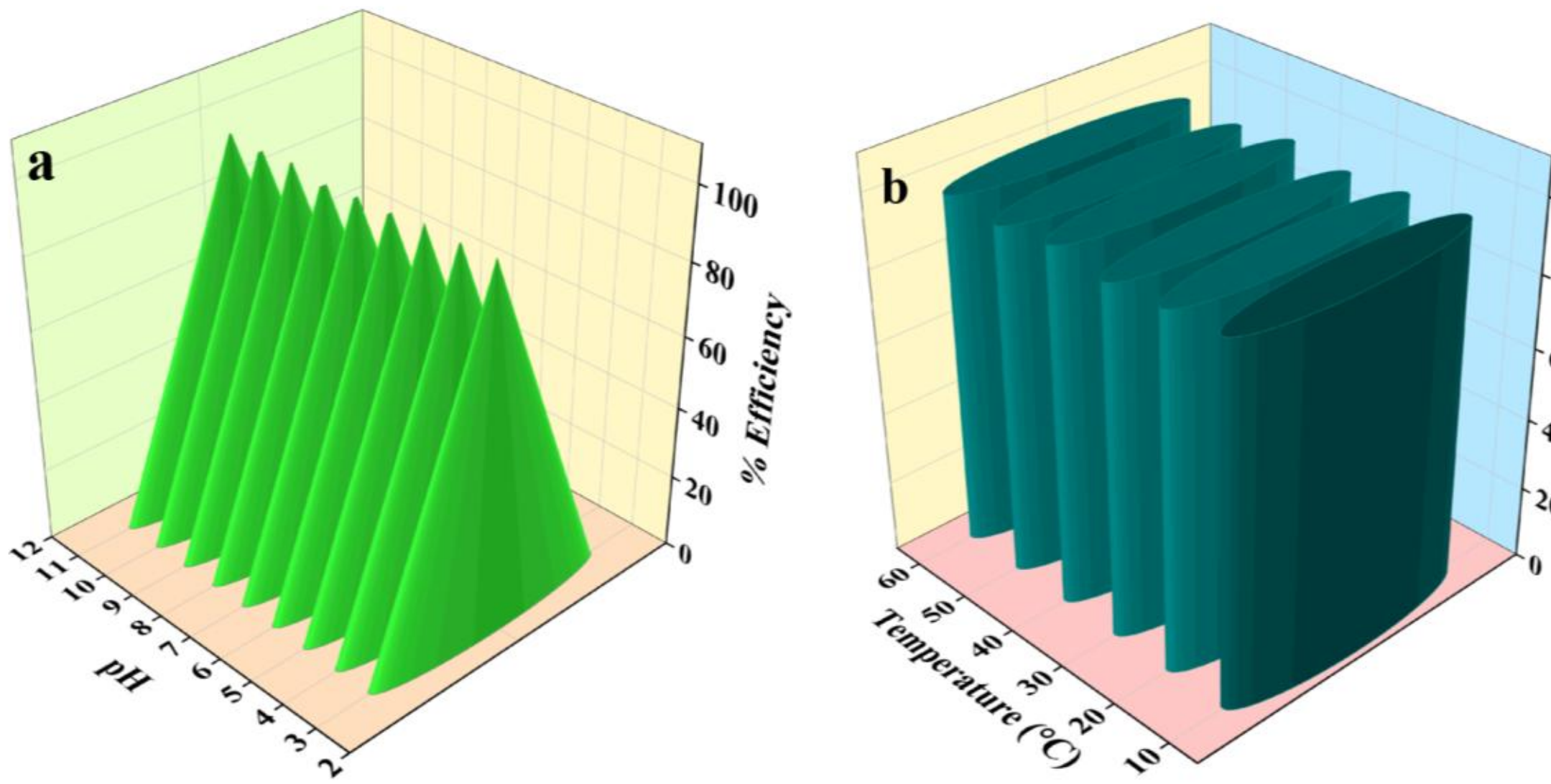

Figure S5: Effect of pH (a) and temperature (b) on quenching response of probe **CPT** toward 4-NT.

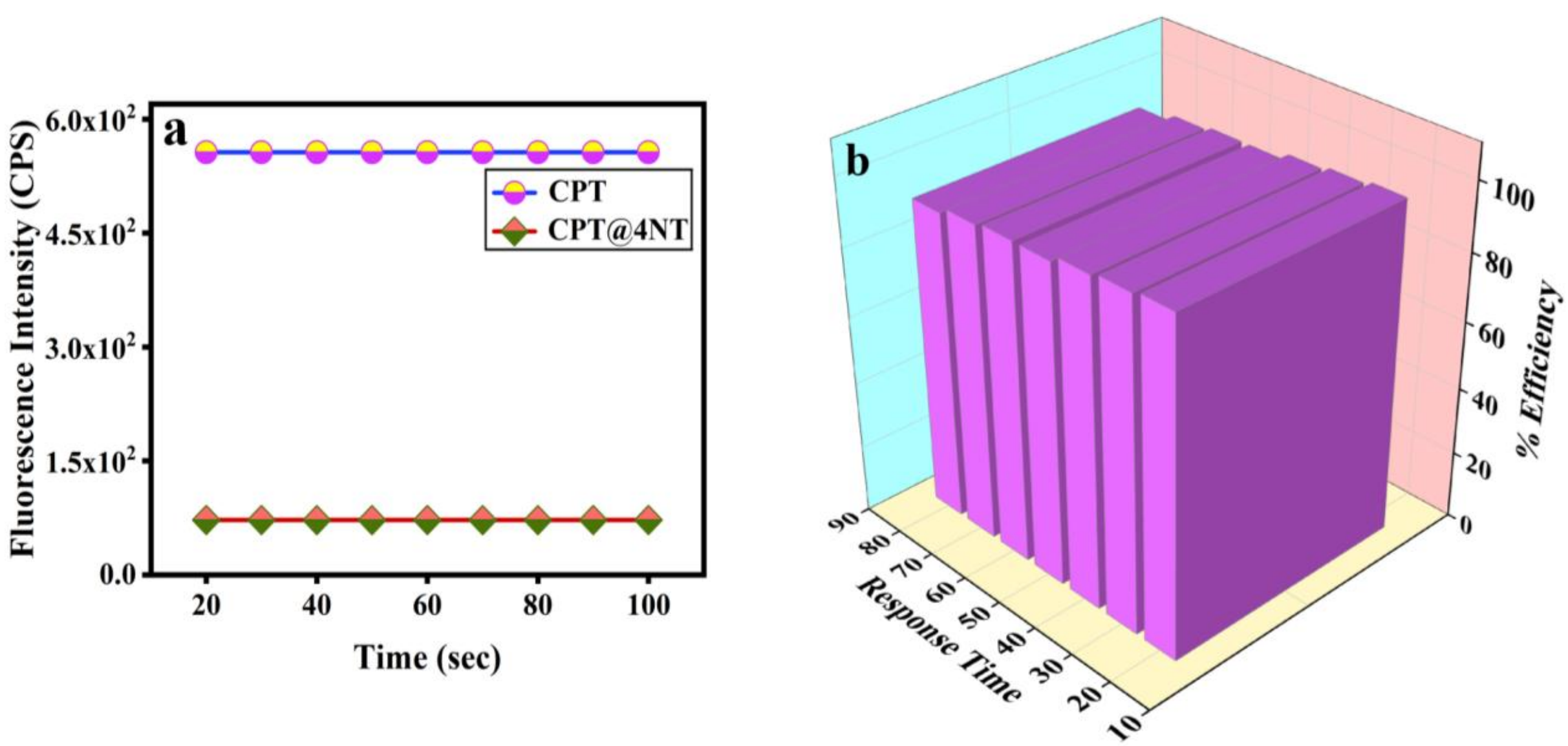

Figure S6: Photostability test of probe **CPT** towards 4-NT (a) and relative fluorescence quenching of probe **CPT** upon exposure to 4-NT within the time interval of 20-80 s (b)

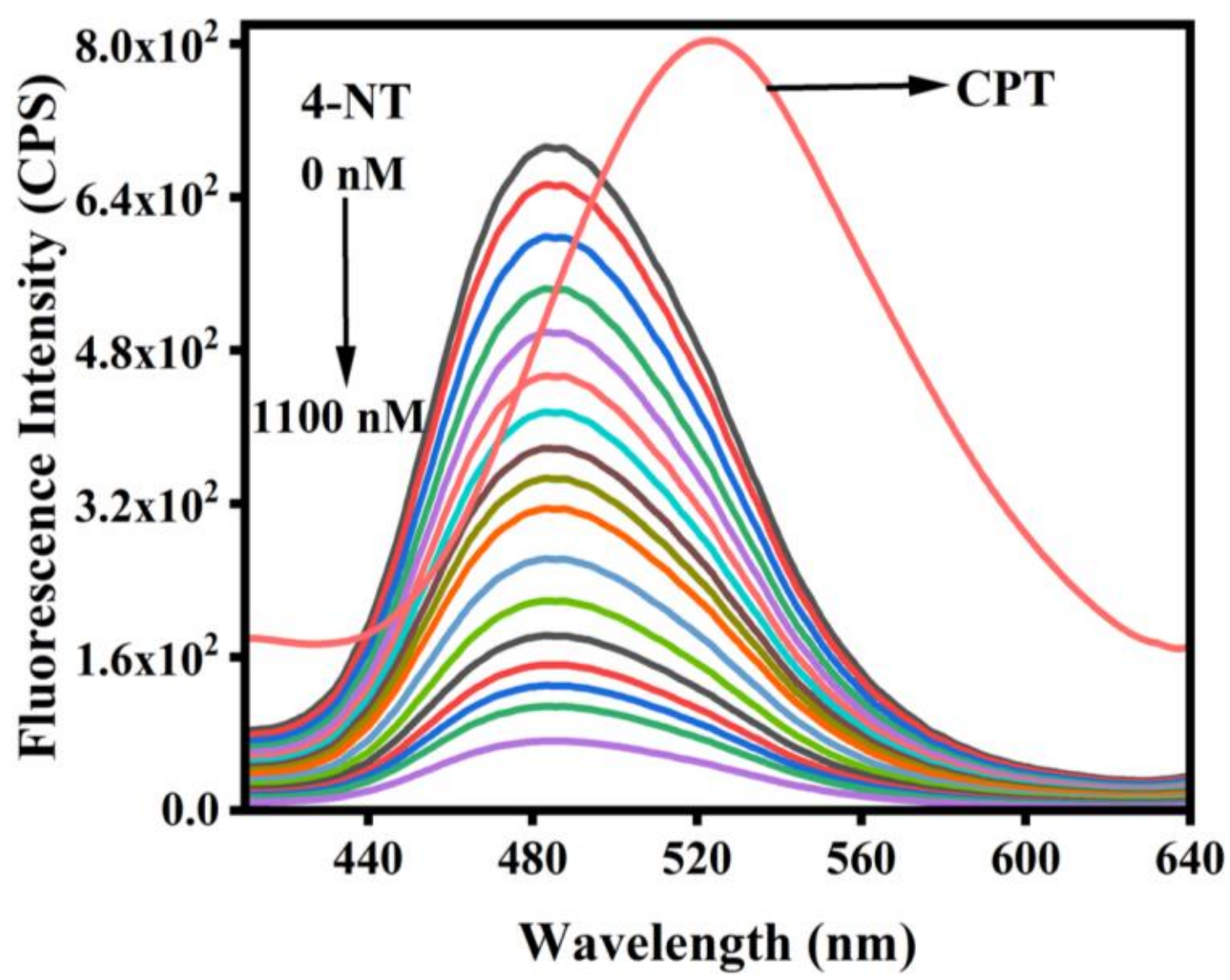

Figure S7: Emission response of probe **CPT** against different concentration of industrial sample

Table S1: The quantum yield of probe CPT with different water fractions

Water Fraction (%)	Quantum Yield
0	0.362
10	0.274
30	0.193
50	0.122
70	0.586
90	0.781

Table S2: Representation of BCPs of CPT@4-NT

BCPs	CPT@4-NT	ρ(r) (a.u)	∇2ρ(r) (a.u)	G(r) (a.u)	V(r) (a.u)	H(r) (a.u)	-V/G	Eint (kcal/mol)
114	(11)C--O(64)	0.0088	0.0318	0.0068	-0.0056	0.0011	-0.8235	-1.757
120	(29)C--C(25)	0.3029	-0.8418	0.0928	-0.3960	-0.3032	-4.2672	-124.245
131	(22)C--C(54)	0.0036	0.0092	0.0019	-0.0016	0.00032	-0.8421	-0.502
144	(25)C--C(54)	0.0059	0.0162	0.0033	-0.0026	0.00071	-0.7878	-0.8157
146	(34)C--H(69)	0.0057	0.0173	0.0035	-0.0027	0.00078	-0.7714	-0.8471
154	(35)H--H(67)	0.0062	0.0204	0.0041	-0.0031	0.0010	-0.7560	-0.9726

Table S3: Recovery experiment for detection of 4-NT in an industrial effluent and real water

Sensing of 4-NT in industrial effluent			Sensing of 4-NT in real water		
**Spiked (nM)**	**Recovery (%)**	**RSD (%)**	**Spiked (nM)**	**Recovery (%)**	**RSD (%)**
**00**	**00**	**00**	**00**	**00**	**00**
**100**	**96.3**	**1.20**	**100**	**98.4**	**1.19**
**300**	**97.4**	**1.23**	**300**	**97.6**	**1.21**
**500**	**97.6**	**1.29**	**500**	**97.3**	**1.24**
**700**	**98.2**	**1.21**	**700**	**98.1**	**1.18**
**900**	**98.5**	**1.31**	**900**	**96.5**	**1.26**
**1100**	**97.3**	**1.24**	**1100**	**98.3**	**1.22**

Complete reference for Gaussian 09

Gaussian 09, Revision D.01, M. J. Frisch, G. W. Trucks, H. B. Schlegel, G. E. Scuseria, M. A. Robb, J. R. Cheeseman, G. Scalmani, V. Barone, B. Mennucci, G. A. Petersson, H. Nakatsuji, M. Caricato, X. Li, H. P. Hratchian, A. F. Izmaylov, J. Bloino, G. Zheng, J. L. Sonnenberg, M. Hada, M. Ehara, K. Toyota, R. Fukuda, J. Hasegawa, M. Ishida, T. Nakajima, Y. Honda, O. Kitao, H. Nakai, T. Vreven, J. A. Montgomery, Jr., J. E. Peralta, F. Ogliaro, M. Bearpark, J. J. Heyd, E. Brothers, K. N. Kudin, V. N. Staroverov, T. Keith, R. Kobayashi, J. Normand, K. Raghavachari, A. Rendell, J. C. Burant, S. S. Iyengar, J. Tomasi, M. Cossi, N. Rega,J. M. Millam, M. Klene, J. E. Knox, J. B. Cross, V. Bakken, C. Adamo, J. Jaramillo,R. Gomperts, R. E. Stratmann, O. Yazyev, A. J. Austin, R. Cammi, C. Pomelli, J. W. Ochterski, R. L. Martin, K. Morokuma, V. G. Zakrzewski, G. A. Voth, P. Salvador, J. J. Dannenberg, S. Dapprich, A. D. Daniels, O. Farkas, J. B. Foresman, J. V. Ortiz, J. Cioslowski, and D. J. Fox, Gaussian, Inc., Wallingford CT, 2013.

References

[1] Kohn, W., Becke, A.D., Parr, R.G. Density functional theory of electronic structure. The Journal of Physical Chemistry. 1996, 100, 12974-80.

[2] Abo Dena, A.S., Muhammad, Z.A., Hassan, W.M. Spectroscopic, DFT studies and electronic properties of novel functionalized bis-1, 3, 4-thiadiazoles. Chemical Papers. 2019, 73, 2803-12.

[3] Christy, P.A., Premkumar, S., Asath, R.M., Mathavan, T., Benial, A.M.F. Vibrational spectroscopic and structural investigations on fullerene: A DFT approach. In: AIP Conference Proceedings, AIP Publishing LLC, 2016, Vol. 1728, p. 020449.

[4] Paizs, B., Suhai, S. Comparative study of BSSE correction methods at DFT and MP2 levels of theory. Journal of Computational Chemistry. 1998, 19, 575-84.

[5] O'boyle, N.M., Tenderholt, A.L., Langner, K.M. Cclib: a library for package-independent computational chemistry algorithms. Journal of Computational Chemistry. 2008, 29, 839-45.

[6] Rafique, S., Alzahrani, A.Y.A., Irshad, H., Khan, A.M., Shahzad, S.A. New fluorescent probe for sensing of mefenamic acid in aqueous medium: An integrated experimental and theoretical analysis. Spectrochimica Acta Part A: Molecular and Biomolecular Spectroscopy. 2023, 300, 122946.

[7] Bader, R.F. Atoms in molecules. Accounts of Chemical Research. 1985, 18, 9-15.

[8] Humphrey, W., Dalke, A., Schulten, K. VMD: Visual molecular dynamics. Journal of Molecular Graphics. 1996, 14, 33-8.

[9] Lu, T., Chen, F. Multiwfn: A multifunctional wavefunction analyzer. Journal of Computational Chemistry. 2012, 33, 580-92.

[10] Williams, T., Kelley, C., Bröker, H., Campbell, J., Cunningham, R., Denholm, D., et al. Gnuplot 4.5: an interactive plotting program. 2011. gnuplot. info. 2017, 56.

Compound 3: 1H NMR (400 MHz, CDCl3)

HK-02/CDCl3/Huda Khalid/ Dr. Sohail A. Shahzad/Dr. Umar Farooq/CUI-ATD(22-3-2025

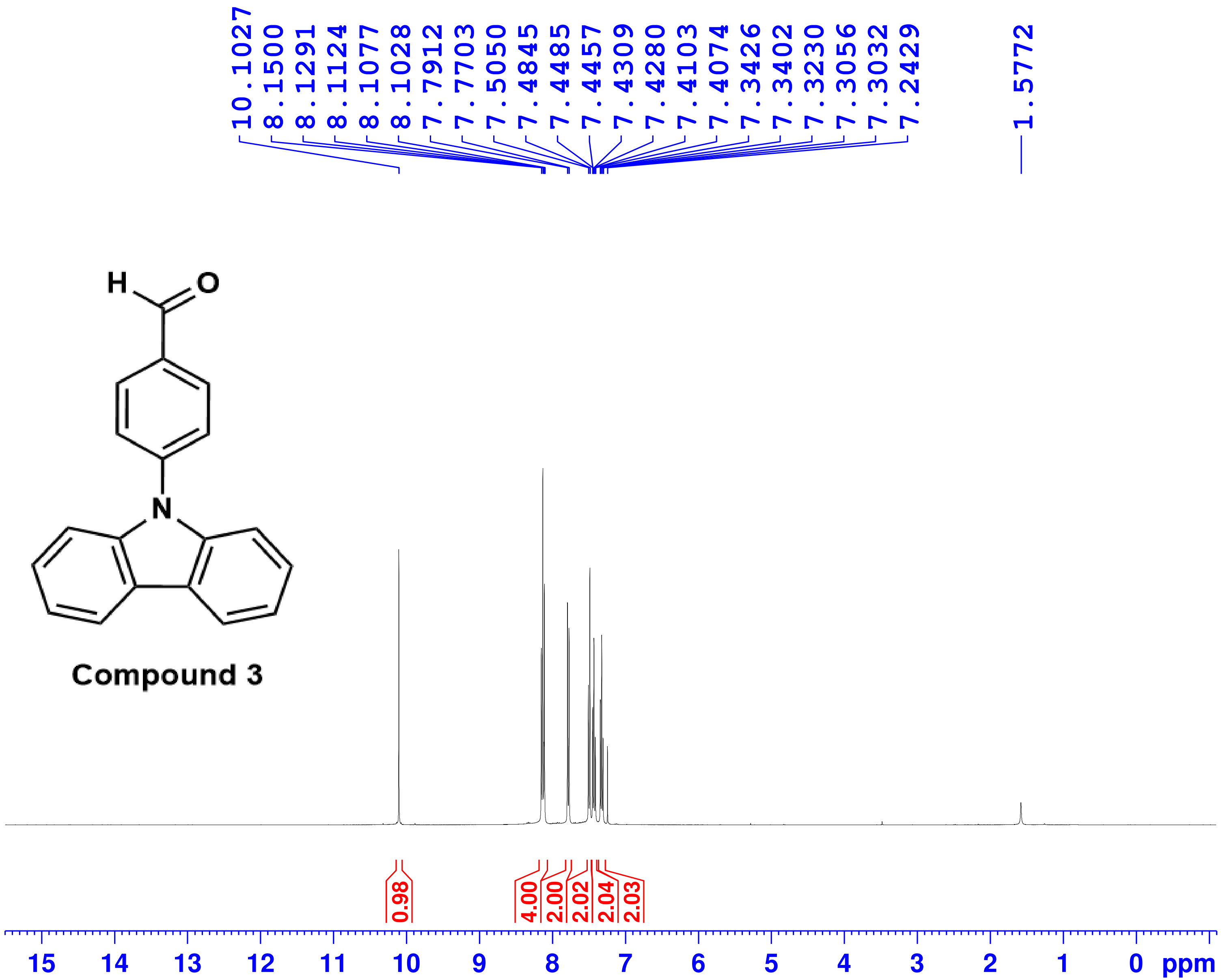

BRUKER

Current Data Parameters
NAME Huda Khalid
EXPNO 199
PROCNO 1

F2 - Acquisition Parameters
Date_ 20250322
Time 13.49 h
INSTRUM spect
PROBHD Z116098_0621 (
PULPROG zg30
TD 65536
SOLVENT CDCl3
NS 16
DS 2
SWH 8012.820 Hz
FIDRES 0.244532 Hz
AQ 4.0894465 sec
RG 88.48
DW 62.400 usec
DE 6.50 usec
TE 298.0 K
D1 1.00000000 sec
TD0 1
SFO1 400.1324708 MHz
NUC1 1H
P1 10.00 usec
PLW1 16.68099976 W

F2 - Processing parameters
SI 65536
SF 400.1300000 MHz
WDW EM
SSB 0
LB 0.30 Hz
GB 0
PC 1.00

Compound 3: 1H NMR (400 MHz, CDCl3)

HK-02/CDCl3/Huda Khalid/ Dr. Sohail A. Shahzad/Dr. Umar Farooq/CUI-ATD(22-3-2025)

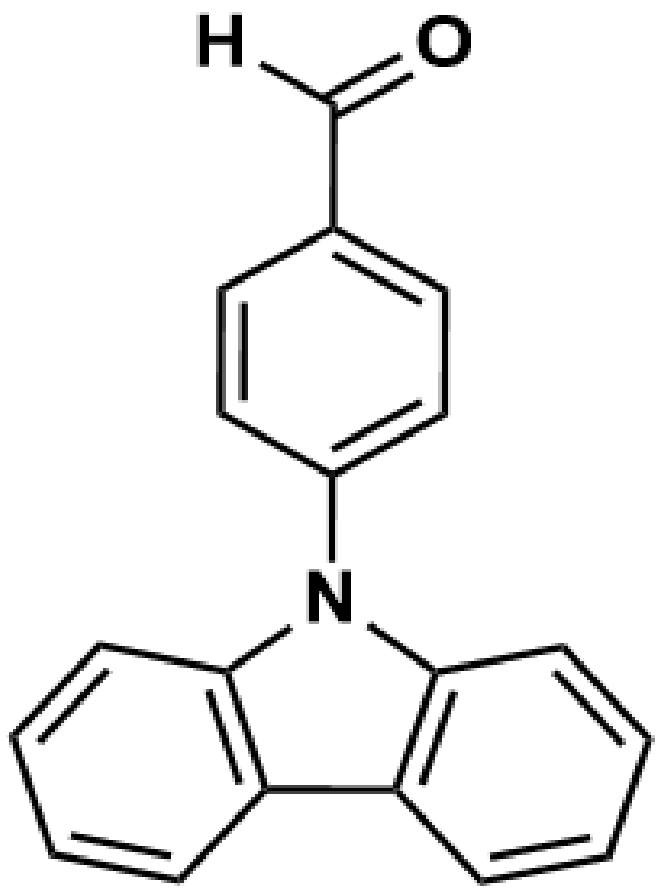

Compound 3

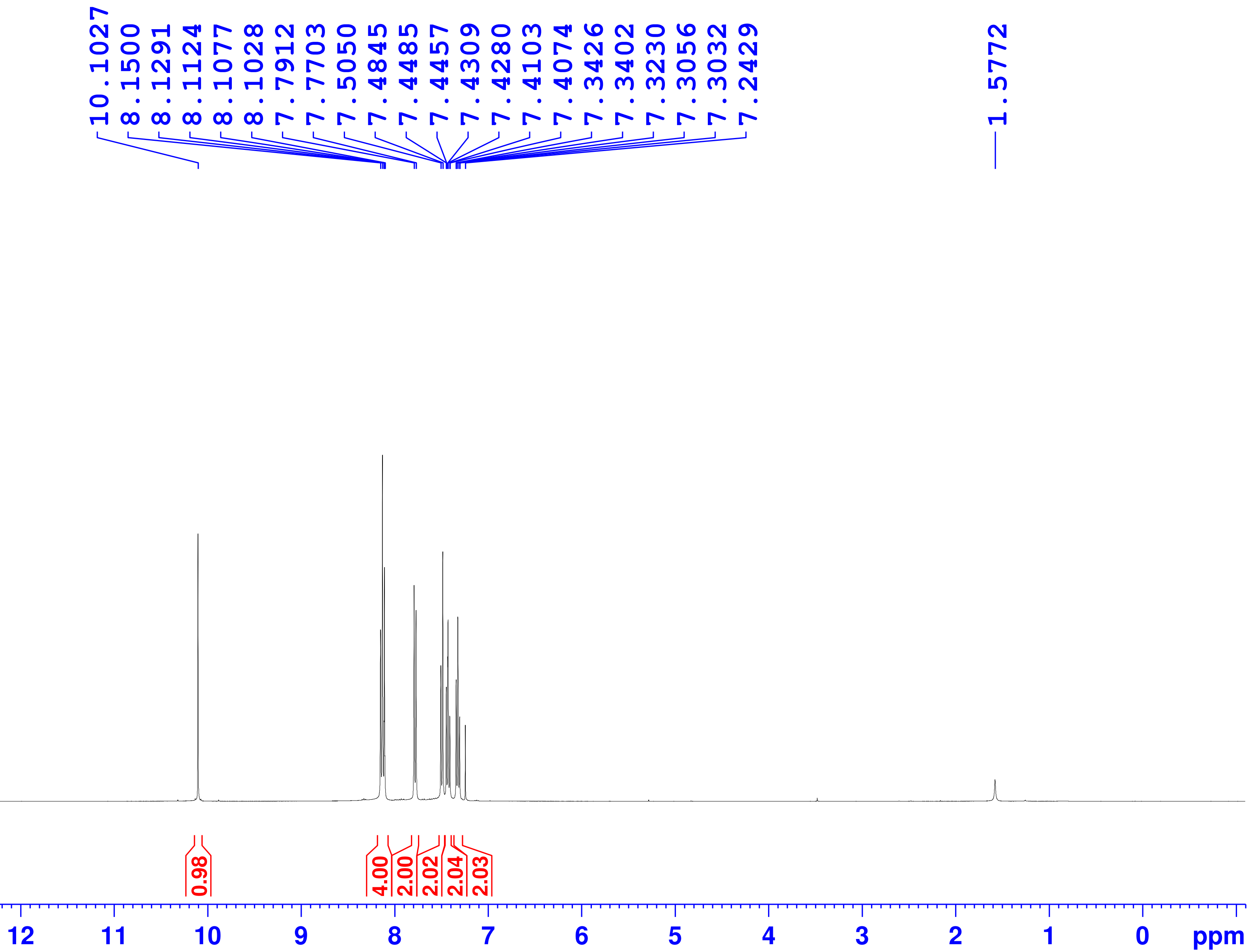

Compound 3: 1H NMR (400 MHz, CDCl3)

HK-02/CDCl3/Huda Khalid/ Dr. Sohail A. Shahzad/Dr. Umar Farooq/CUI-ATD(22-3-2025)

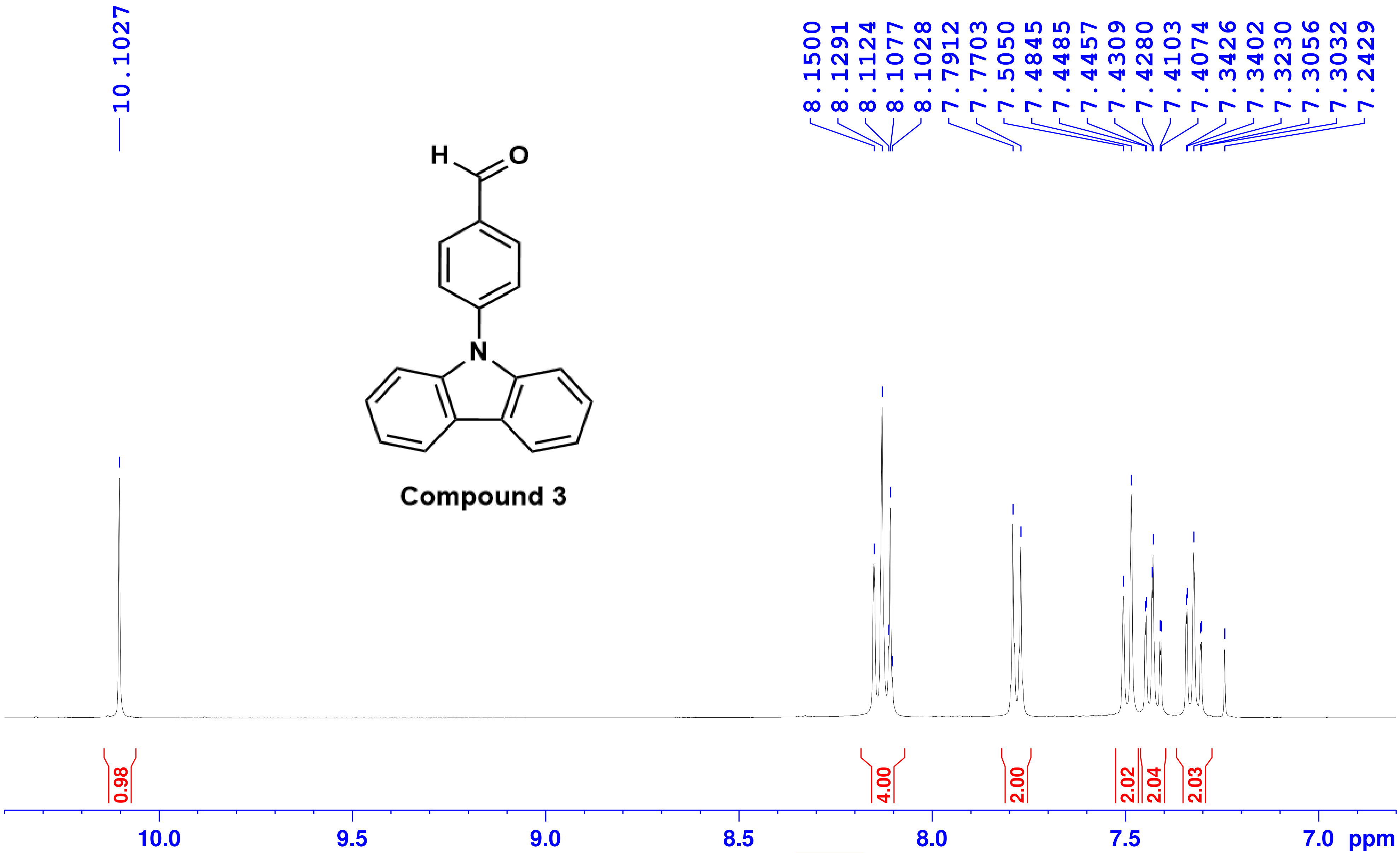

Compound 3: 1H NMR (400 MHz, CDCl3)

HK-02/CDCl3/Huda Khalid/ Dr. Sohail A. Shahzad/Dr. Umar Farooq/CUI-ATD(22-3-2025)

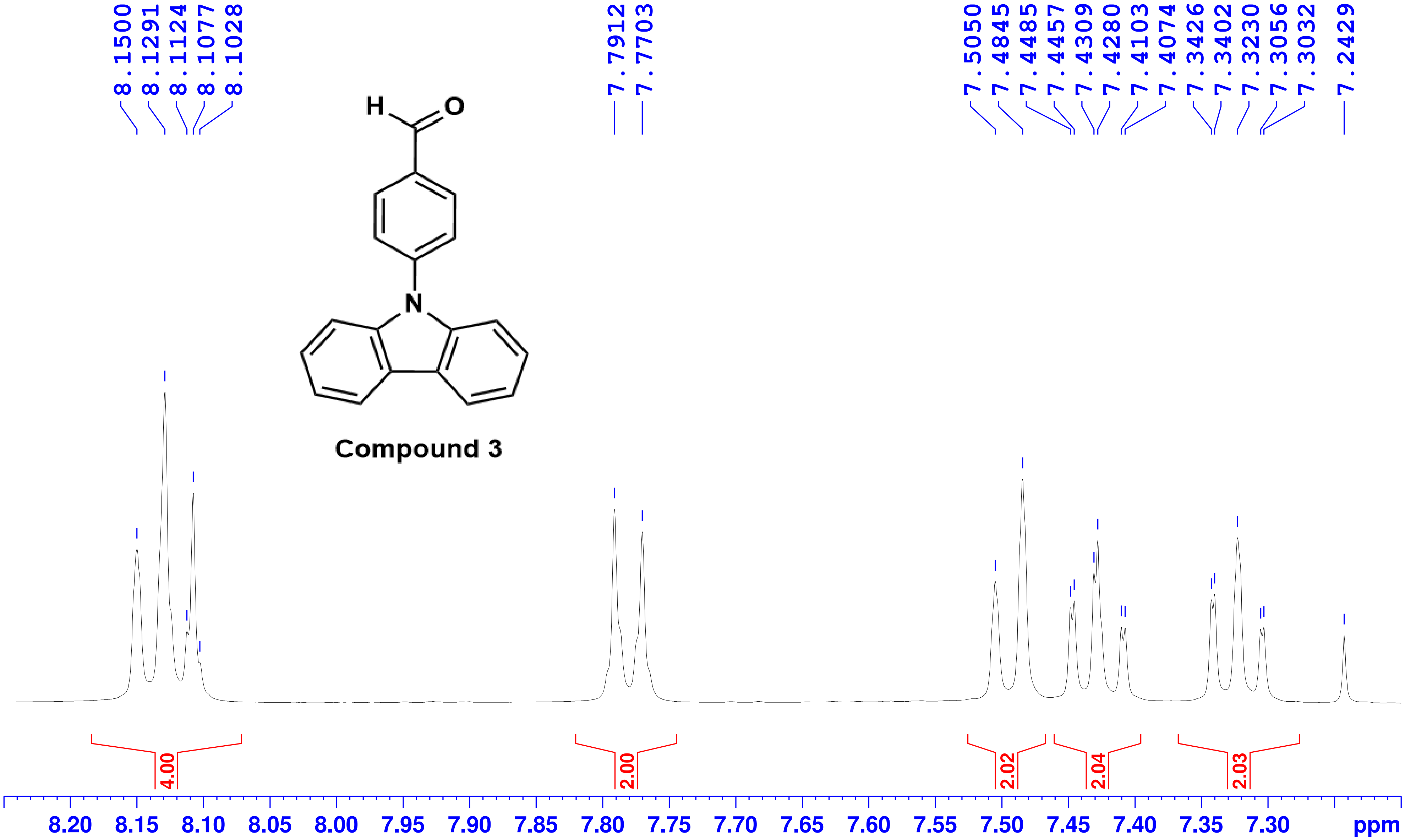

Compound 3

Compound 3: 1H NMR (400 MHz, CDCl3)

HK-02/CDCl3/Huda Khalid/ Dr. Sohail A. Shahzad/Dr. Umar Farooq/CUI-ATD(22-3-2025)

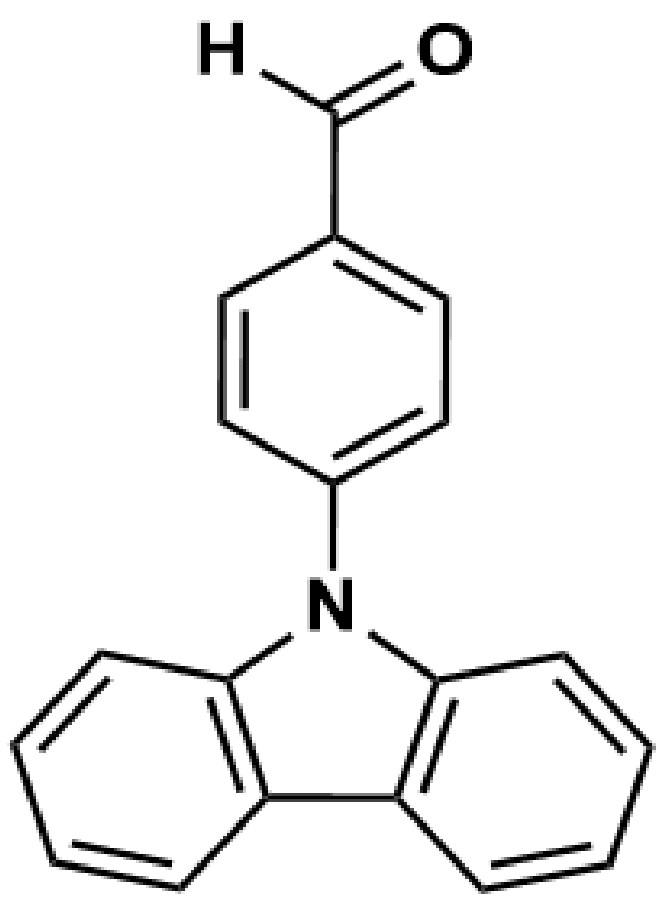

Compound 3

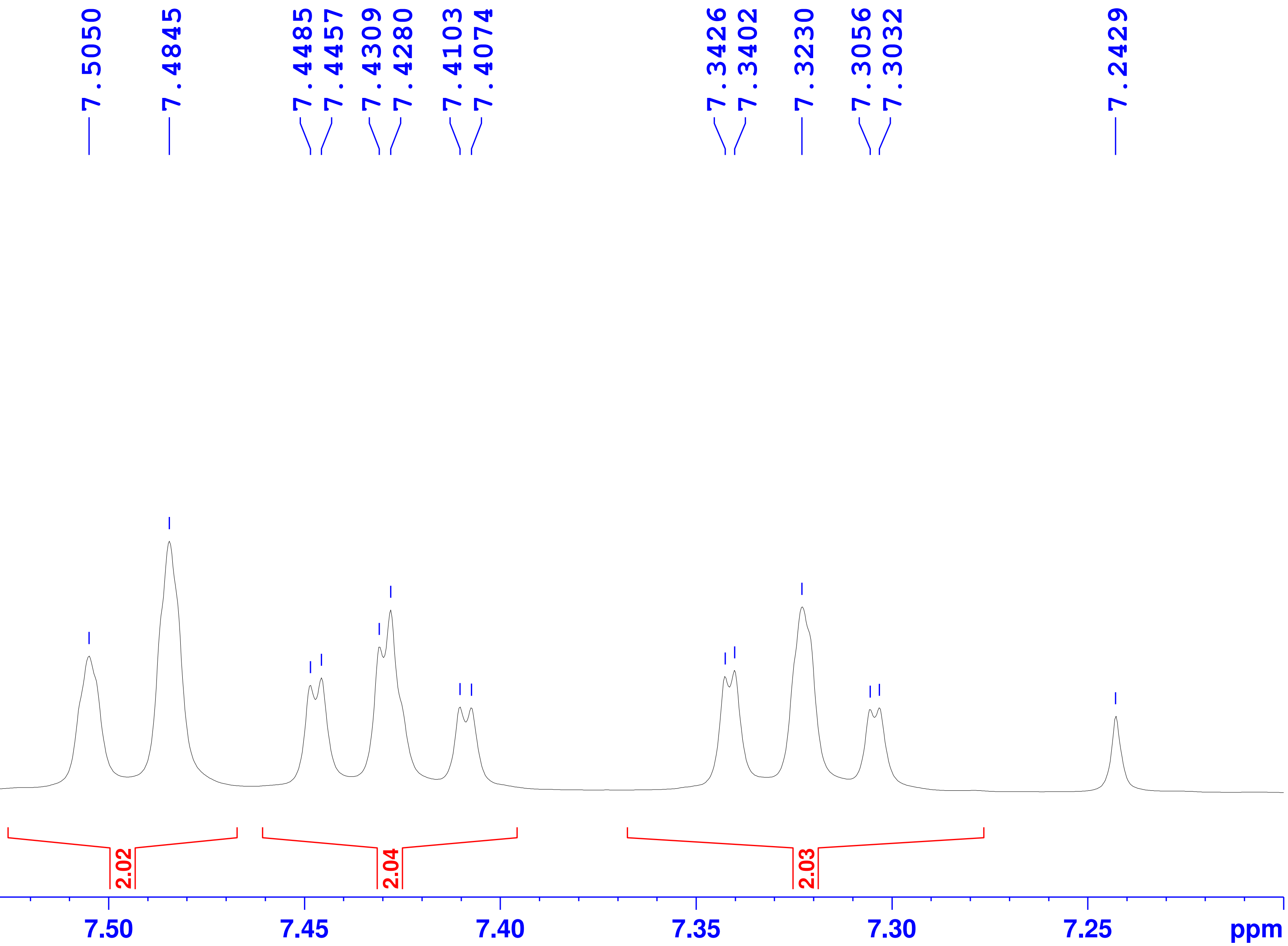

Compound 3: 13C NMR (100 MHz, CDCl3)

HK-02/DMSO-d6/Huda Khalid/ Dr. Umar Farooq/CUI-ATD (22-3-2025)

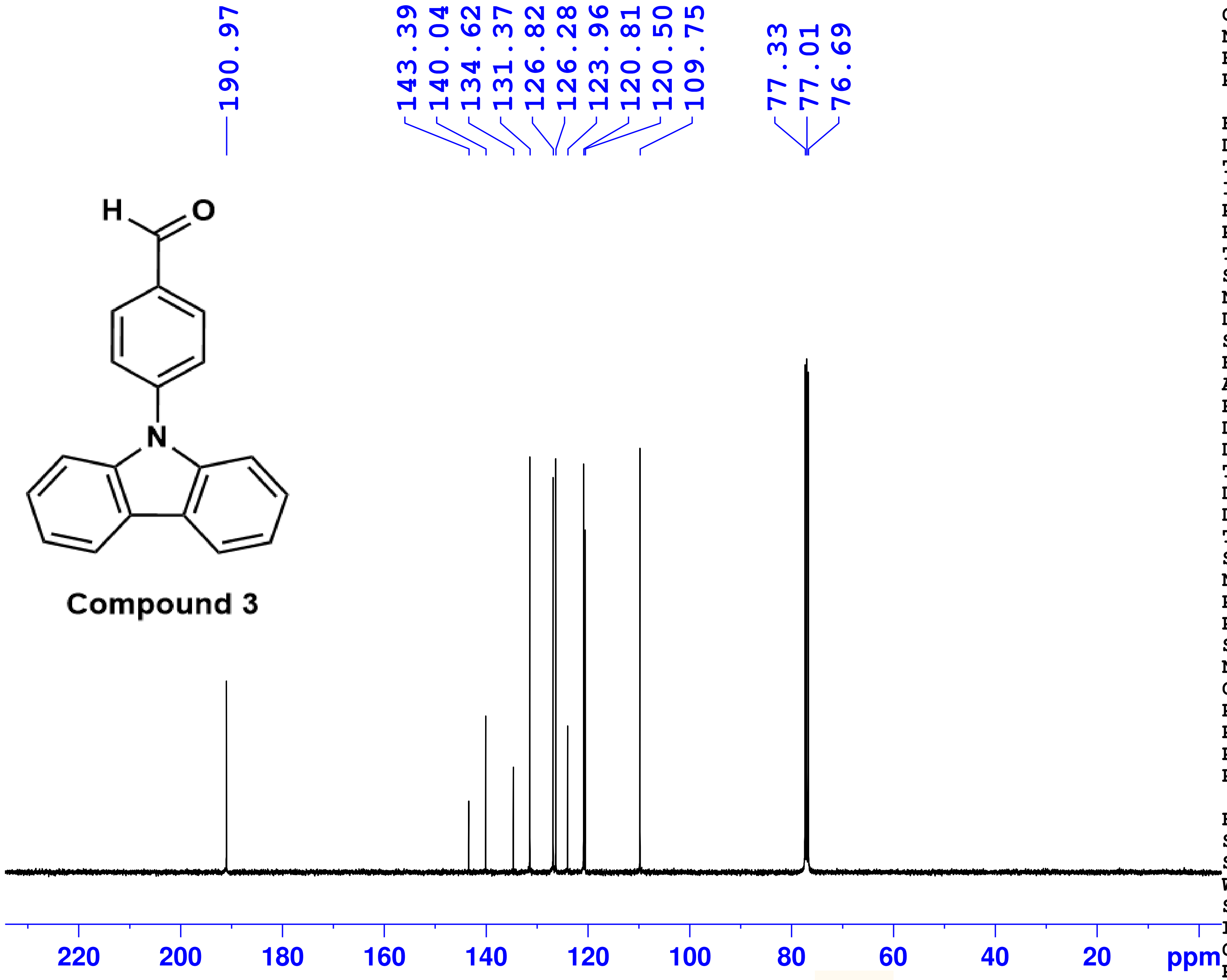

Current Data Parameters

NAME	Huda Khalid
EXPNO	204
PROCNO	1

F2 - Acquisition Parameters

Date_	20250322
Time	18.58 h
INSTRUM	spect
PROBHD	Z116098_0621 (
PULPROG	zgpg30
TD	65536
SOLVENT	CDCl3
NS	1024
DS	2
SWH	24038.461 Hz
FIDRES	0.733596 Hz
AQ	1.3631488 sec
RG	199.48
DW	20.800 usec
DE	6.50 usec
TE	298.0 K
D1	2.00000000 sec
D11	0.03000000 sec
TD0	1
SFO1	100.6243390 MHz
NUC1	13C
P1	10.00 usec
PLW1	72.56700134 W
SFO2	400.1316005 MHz
NUC2	1H
CPDPRG[2	waltz16
PCPD2	90.00 usec
PLW2	16.68099976 W
PLW12	0.20593999 W
PLW13	0.10342000 W

F2 - Processing parameters

SI	32768
SF	100.6127685 MHz
WDW	EM
SSB	0
LB	1.00 Hz
GB	0
PC	1.40

Compound 3: 13C NMR (100 MHz, CDCl3)

HK-02/DMSO-d6/Huda Khalid/ Dr. Umar Farooq/CUI-ATD (22-3-2025)

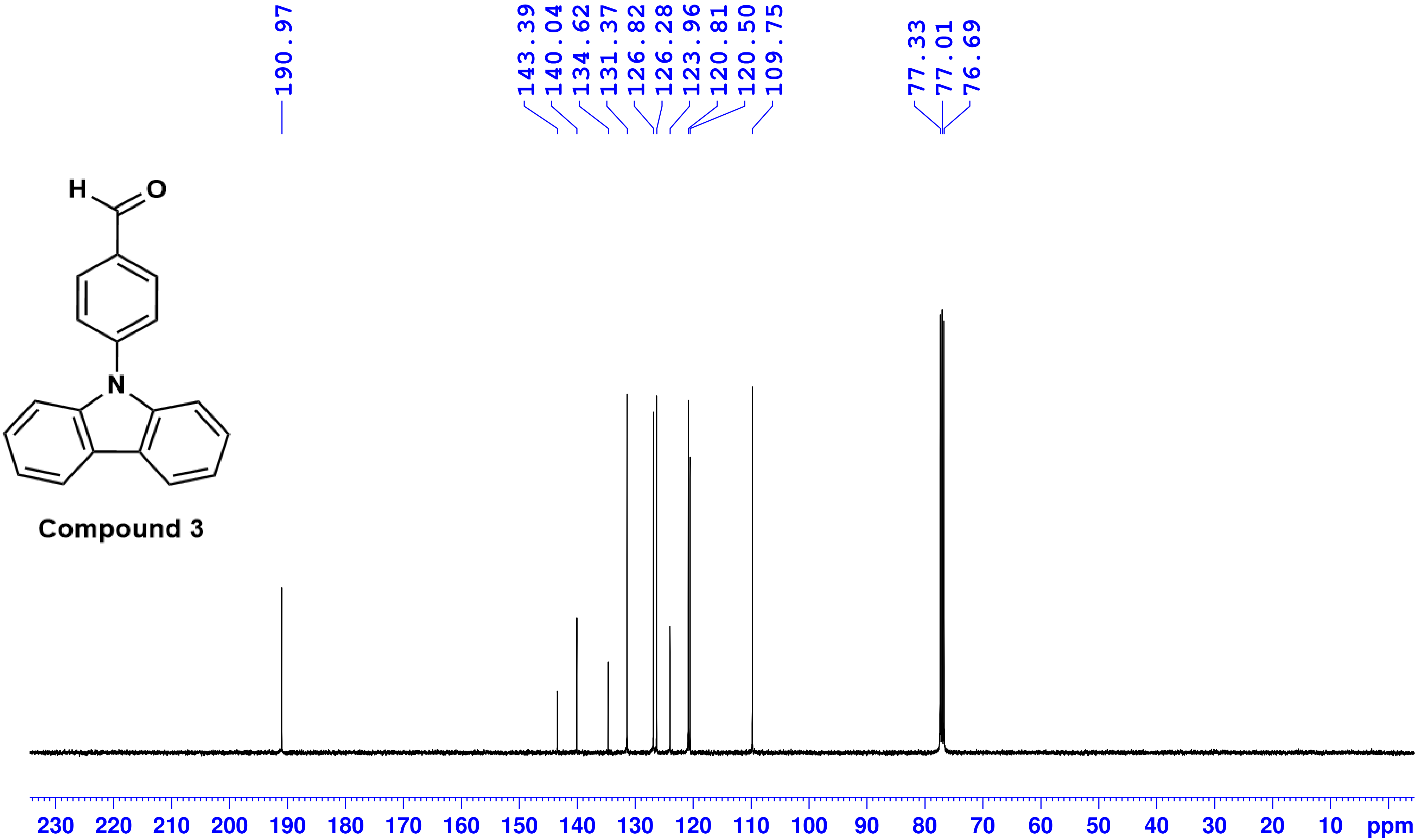

Compound 3: 13C NMR (100 MHz, CDCl3)

HK-02/DMSO-d6/Huda Khalid/ Dr. Umar Farooq/CUI-ATD (22-3-2025)

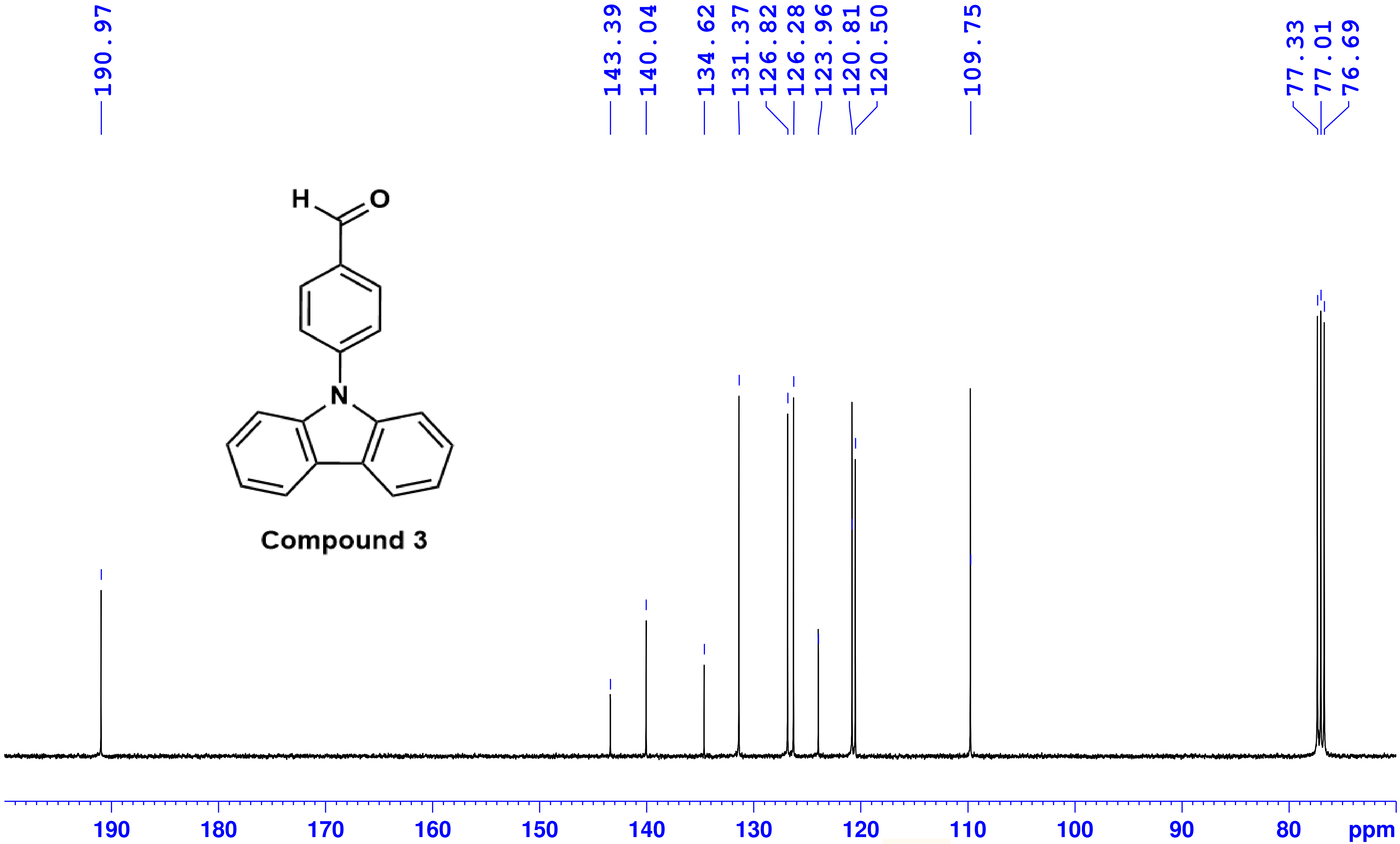

Compound 3: 13C NMR (100 MHz, CDCl3)

HK-02/DMSO-d6/Huda Khalid/ Dr. Umar Farooq/CUI-ATD (22-3-2025)

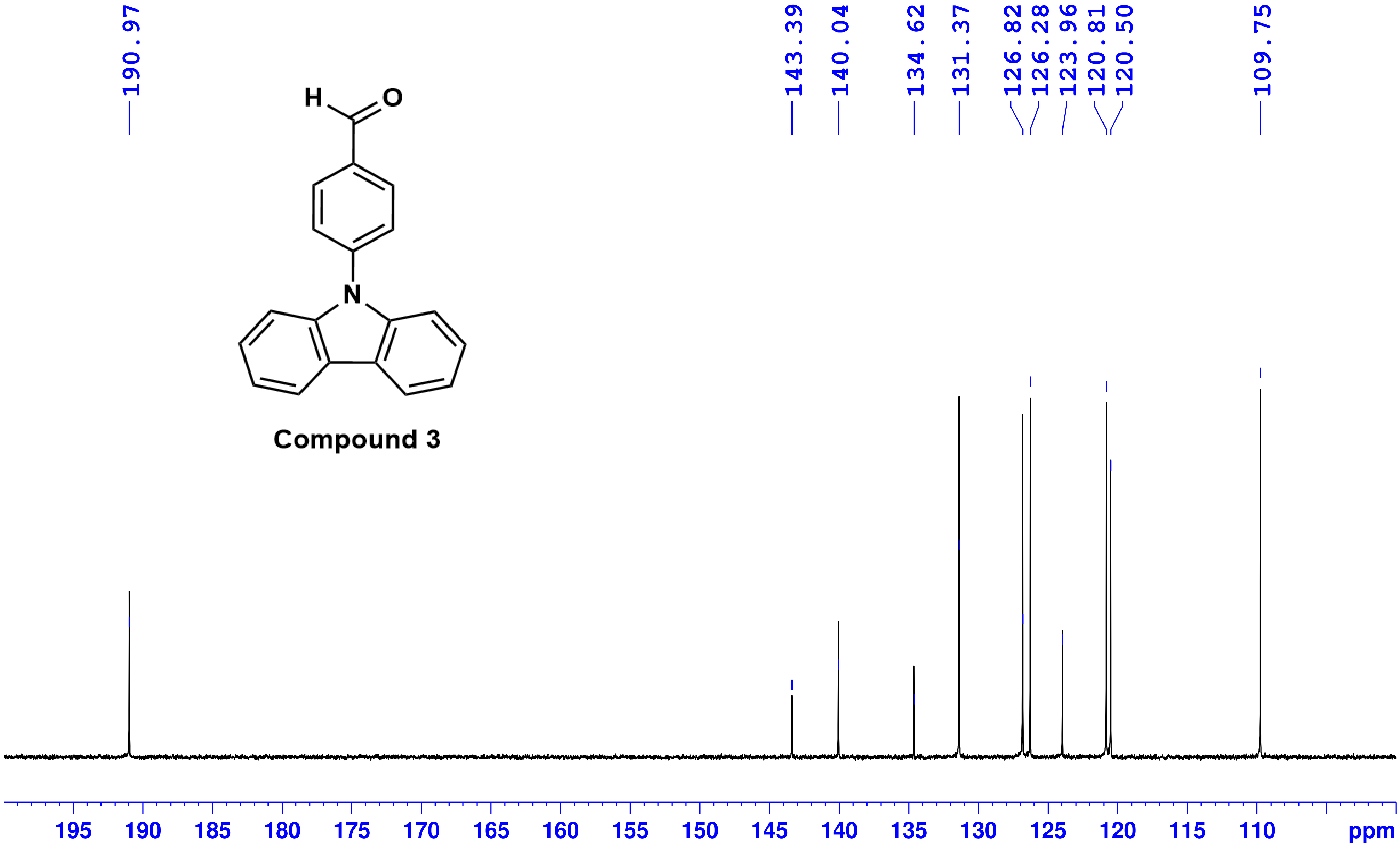

Compound 3: 13C NMR (100 MHz, CDCl3)

HK-02/DMSO-d6/Huda Khalid/ Dr. Umar Farooq/CUI-ATD (22-3-2025)

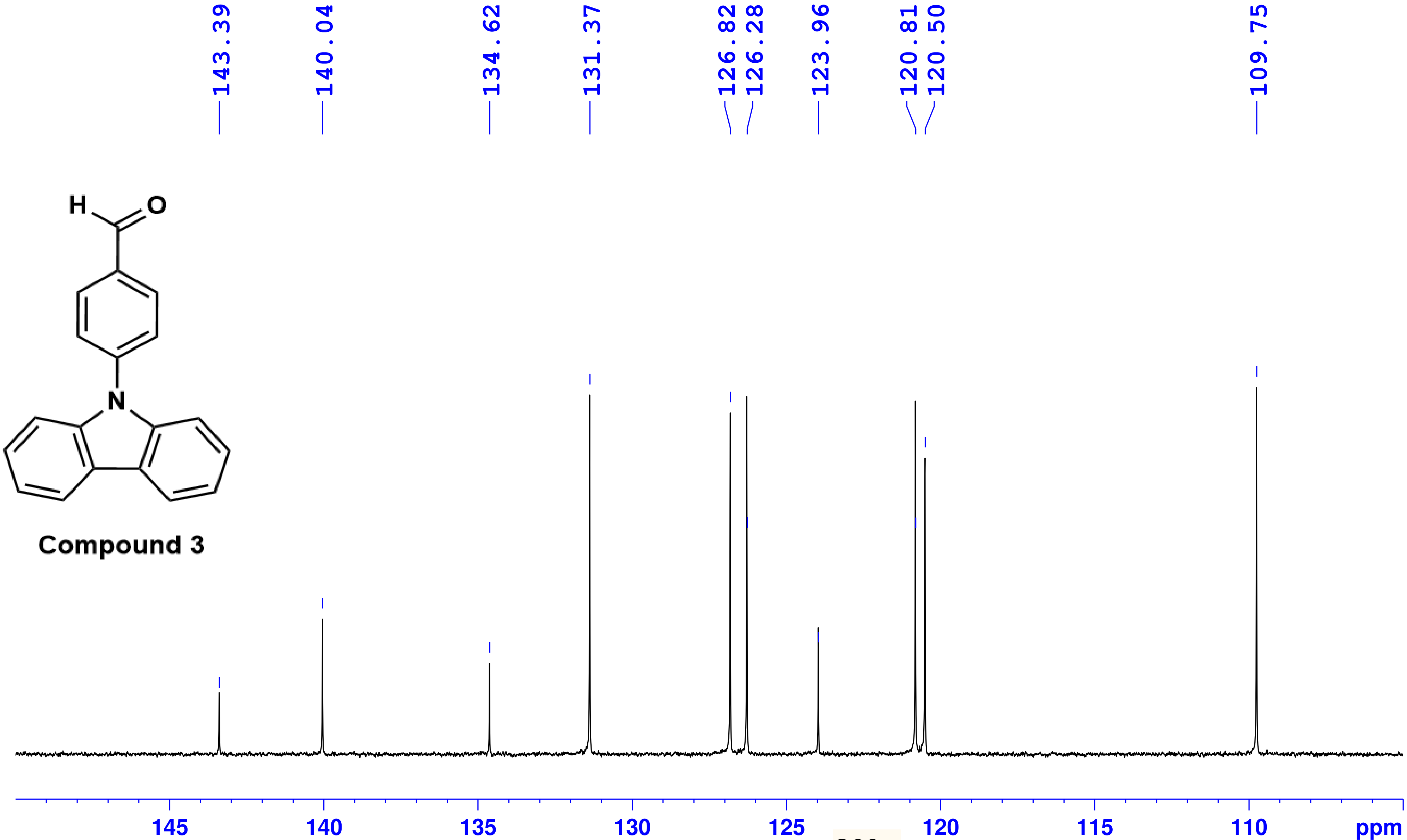

Compound 3

CPT Probe: 1H NMR (400 MHz, CDCl3)

HK-03/CDCl3/Huda Khalid/Dr. Umar Farooq CUI ATD(17-4-2025)

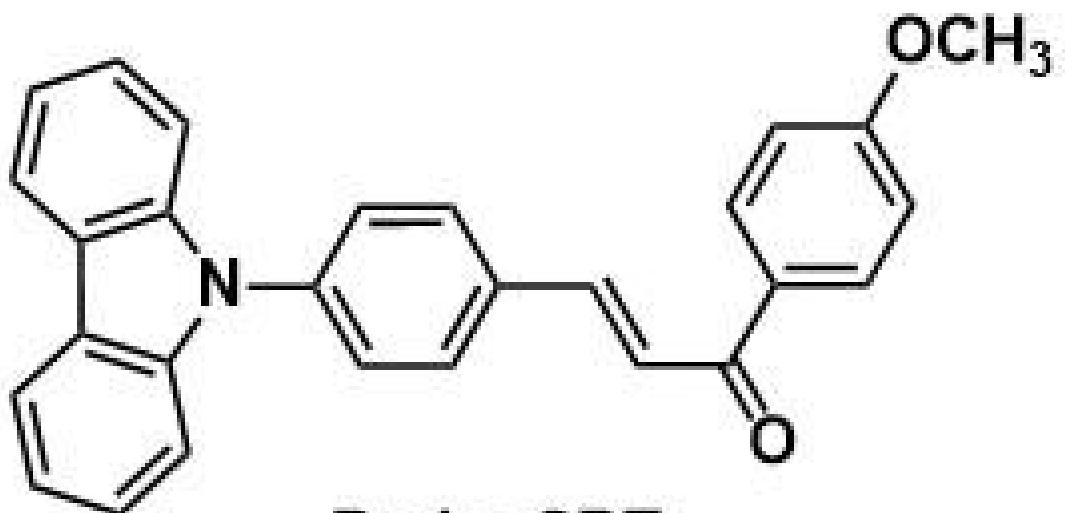

Probe CPT

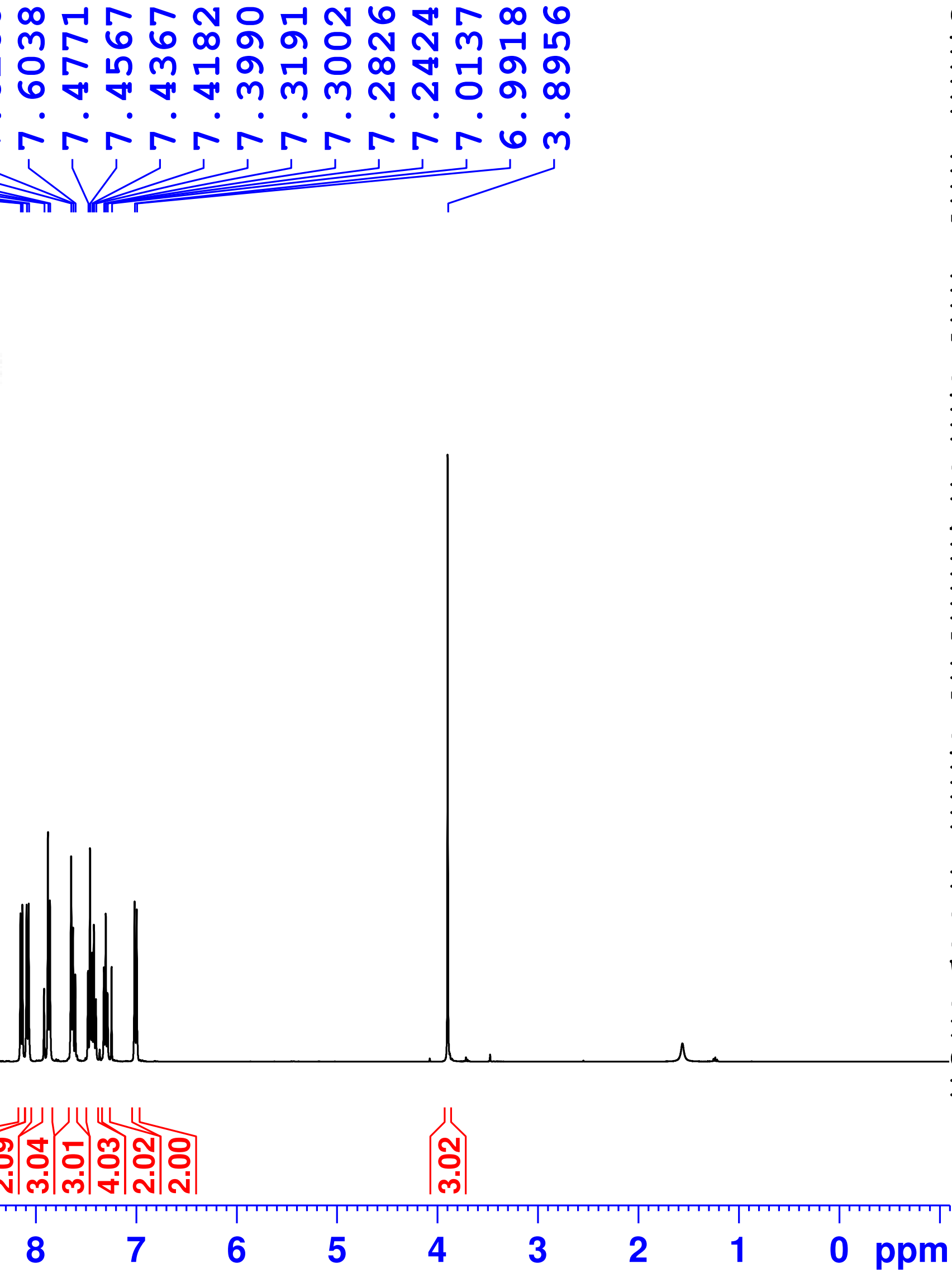

Current Data Parameters	
NAME	Huda Khalid
EXPNO	11
PROCNO	1

F2 - Acquisition Parameters	
Date_	20250417
Time	13.56 h
INSTRUM	spect
PROBHD	Z116098_0621 (
PULPROG	zg30
TD	65536
SOLVENT	CDCl3
NS	32
DS	2
SWH	8012.820 Hz
FIDRES	0.244532 Hz
AQ	4.0894465 sec
RG	112.2
DW	62.400 usec
DE	6.50 usec
TE	298.0 K
D1	1.00000000 sec
TD0	1
SFO1	400.1324708 MHz
NUC1	1H
P1	10.00 usec
PLW1	16.68099976 W

F2 - Processing parameters	
SI	65536
SF	400.1300000 MHz
WDW	EM
SSB	0
LB	0.30 Hz
GB	0
PC	1.00

CPT Probe: 1H NMR (400 MHz, CDCl3)

HK-03/CDCl3/Huda Khalid/Dr. Umar Farooq CUI ATD(17-4-2025)

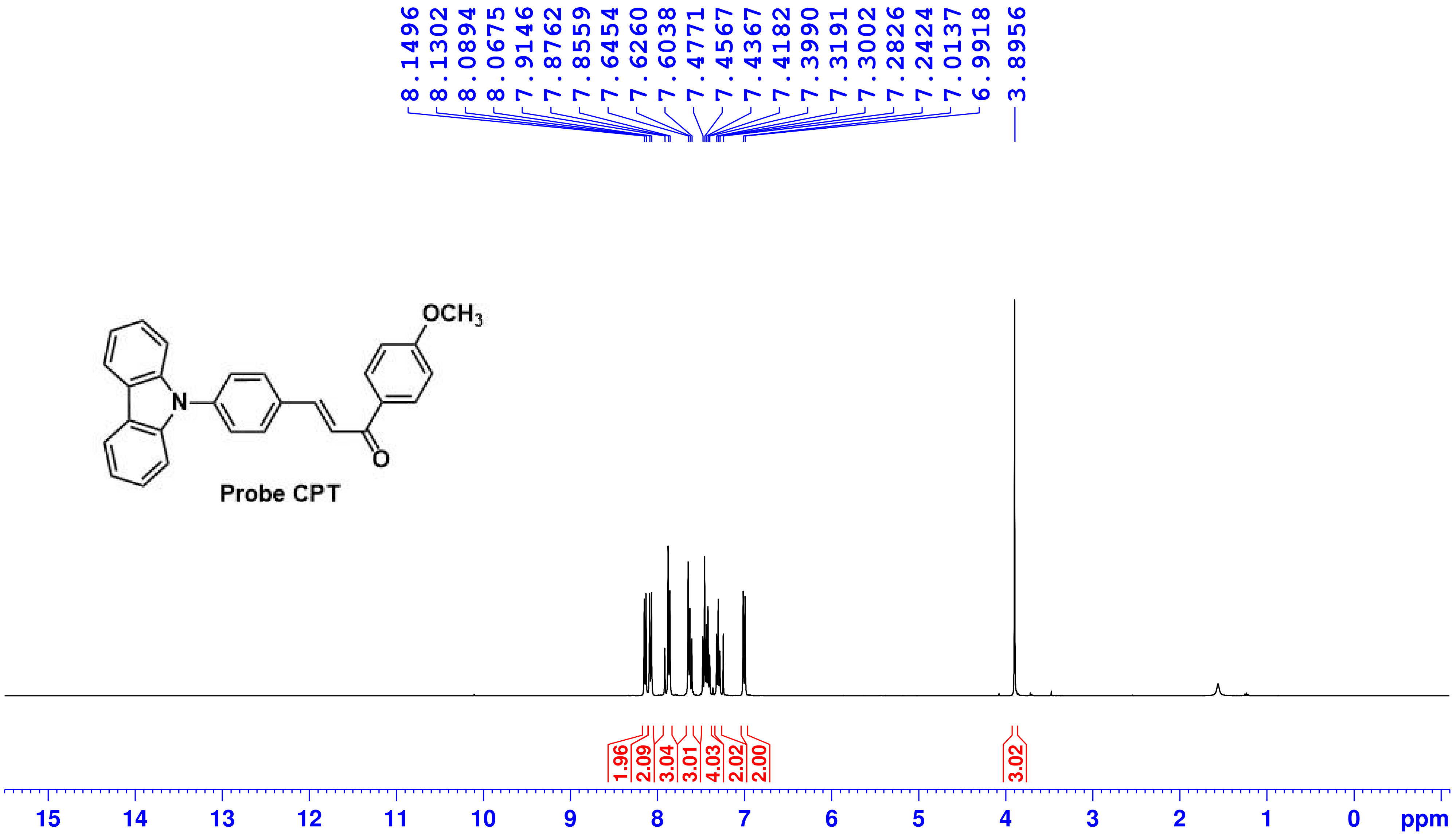

CPT Probe: 1H NMR (400 MHz, CDCl3)

HK-03/CDCl3/Huda Khalid/Dr. Umar Farooq CUI ATD(17-4-2025)

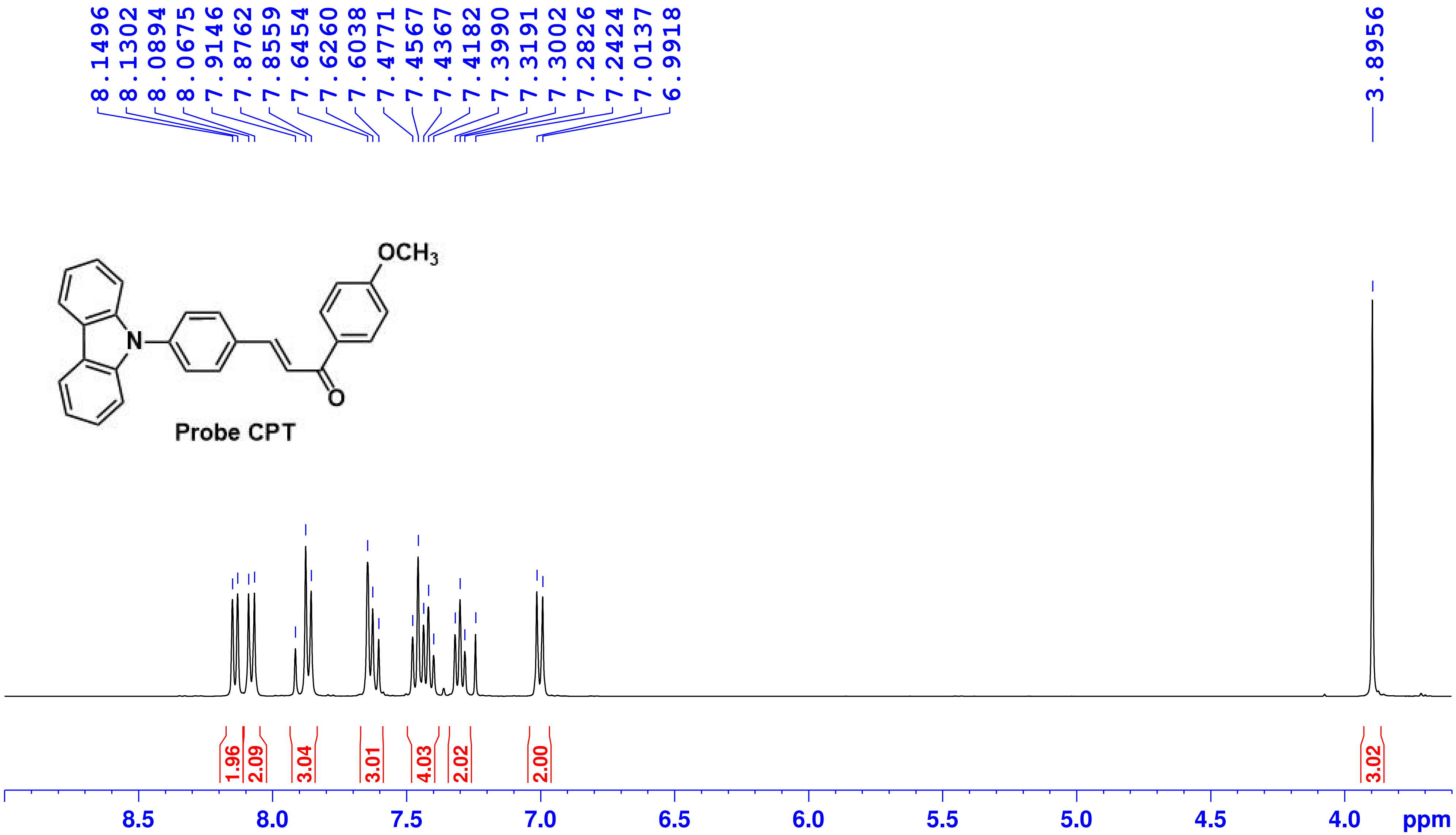

CPT Probe: 1H NMR (400 MHz, CDCl3)

HK-03/CDCl3/Huda Khalid/Dr. Umar Farooq CUI ATD(17-4-2025)

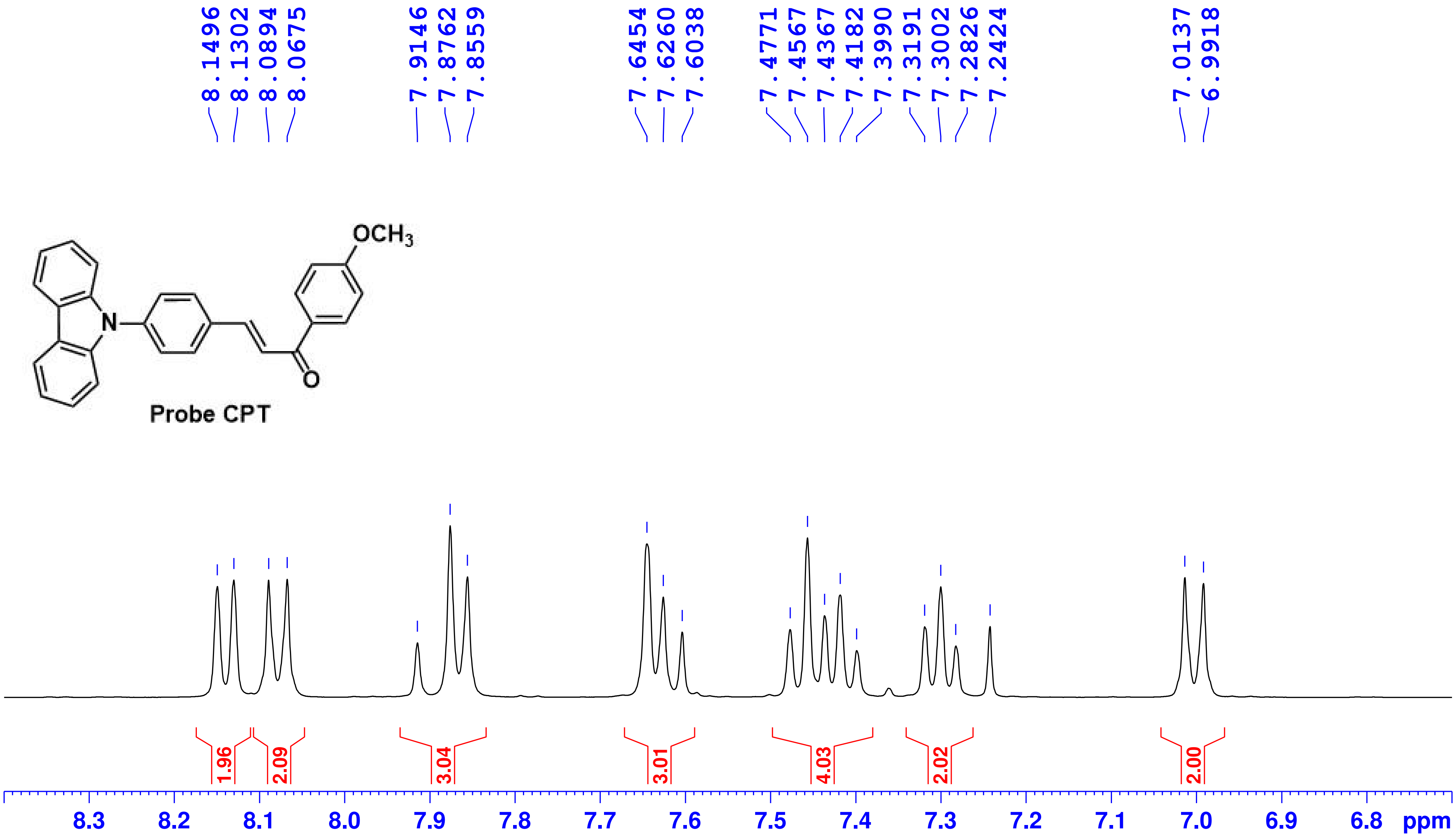

CPT Probe: 13C NMR (100 MHz, CDCl3)

HK-03/CDCl3/Huda Khalid/Dr. Umar Farooq CUI ATD(17-4-2025)

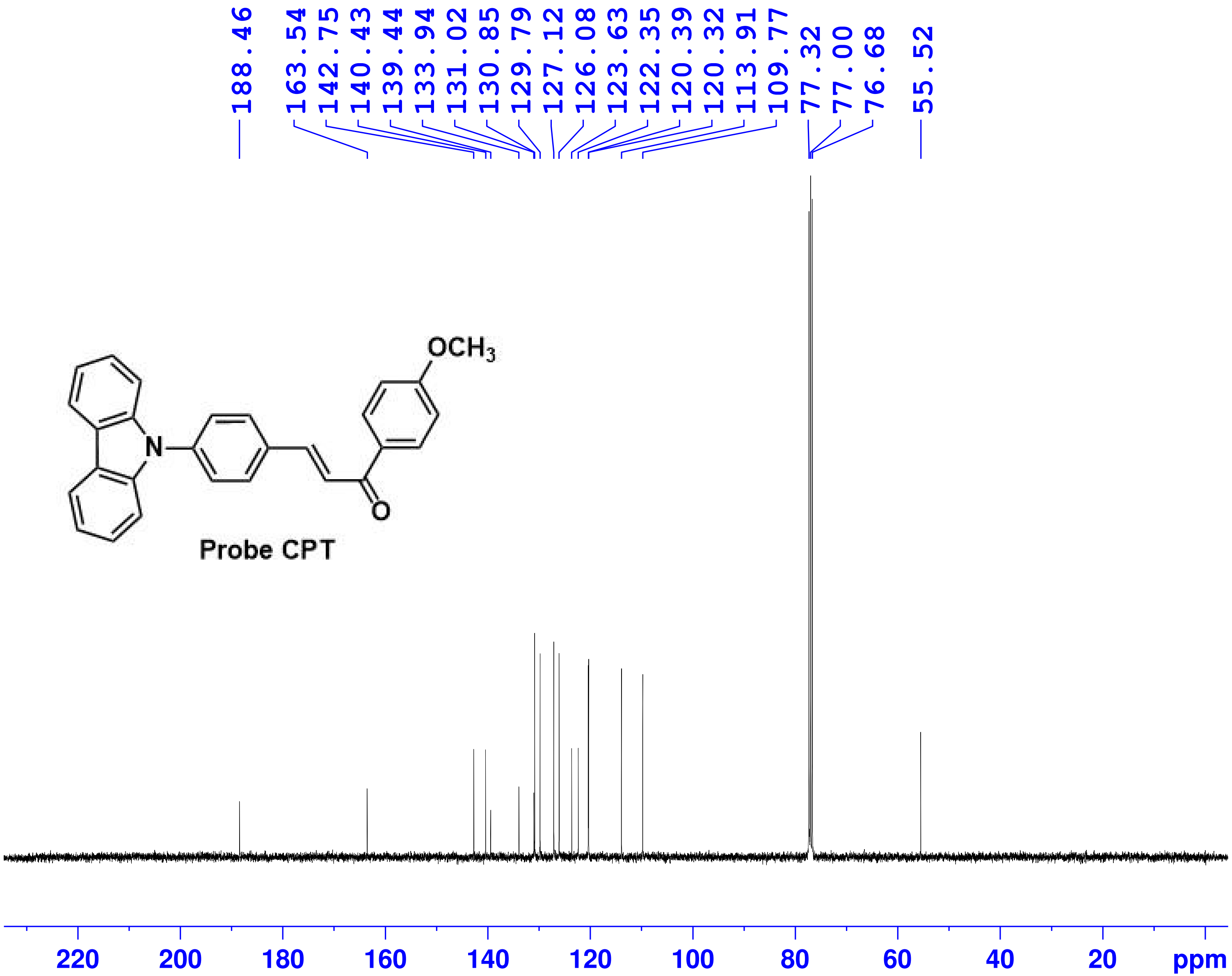

BRUKER

Current Data Parameters

NAME	Huda Khalid
EXPNO	14
PROCNO	1

F2 - Acquisition Parameters

Date_	20250421
Time	13.05 h
INSTRUM	spect
PROBHD	Z116098_0621 (
PULPROG	zgpg30
TD	65536
SOLVENT	CDCl3
NS	400
DS	2
SWH	24038.461 Hz
FIDRES	0.733596 Hz
AQ	1.3631488 sec
RG	199.48
DW	20.800 usec
DE	6.50 usec
TE	298.0 K
D1	2.00000000 sec
D11	0.03000000 sec
TD0	1
SFO1	100.6243390 MHz
NUC1	13C
P1	10.00 usec
PLW1	72.56700134 W
SFO2	400.1316005 MHz
NUC2	1H
CPDPRG[2	waltz16
PCPD2	90.00 usec
PLW2	16.68099976 W
PLW12	0.20593999 W
PLW13	0.10342000 W

F2 - Processing parameters

SI	32768
SF	100.6127685 MHz
WDW	EM
SSB	0
LB	1.00 Hz
GB	0
PC	1.40

CPT Probe: 13C NMR (100 MHz, CDCl3)

HK-03/CDCl3/Huda Khalid/Dr. Umar Farooq CUI ATD(17-4-2025)

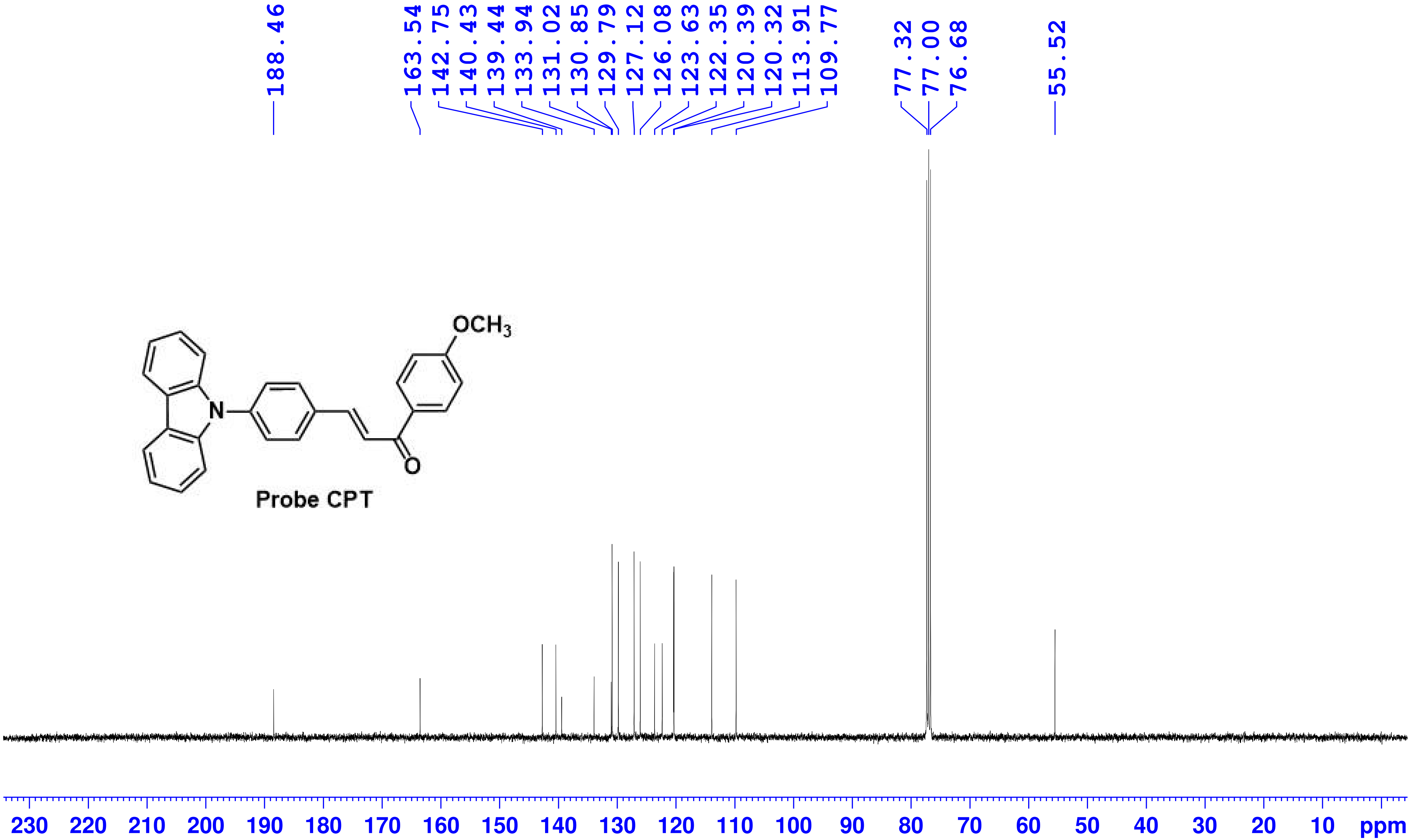

CPT Probe: 13C NMR (100 MHz, CDCl3)

HK-03/CDCl3/Huda Khalid/Dr. Umar Farooq CUI ATD(17-4-2025)

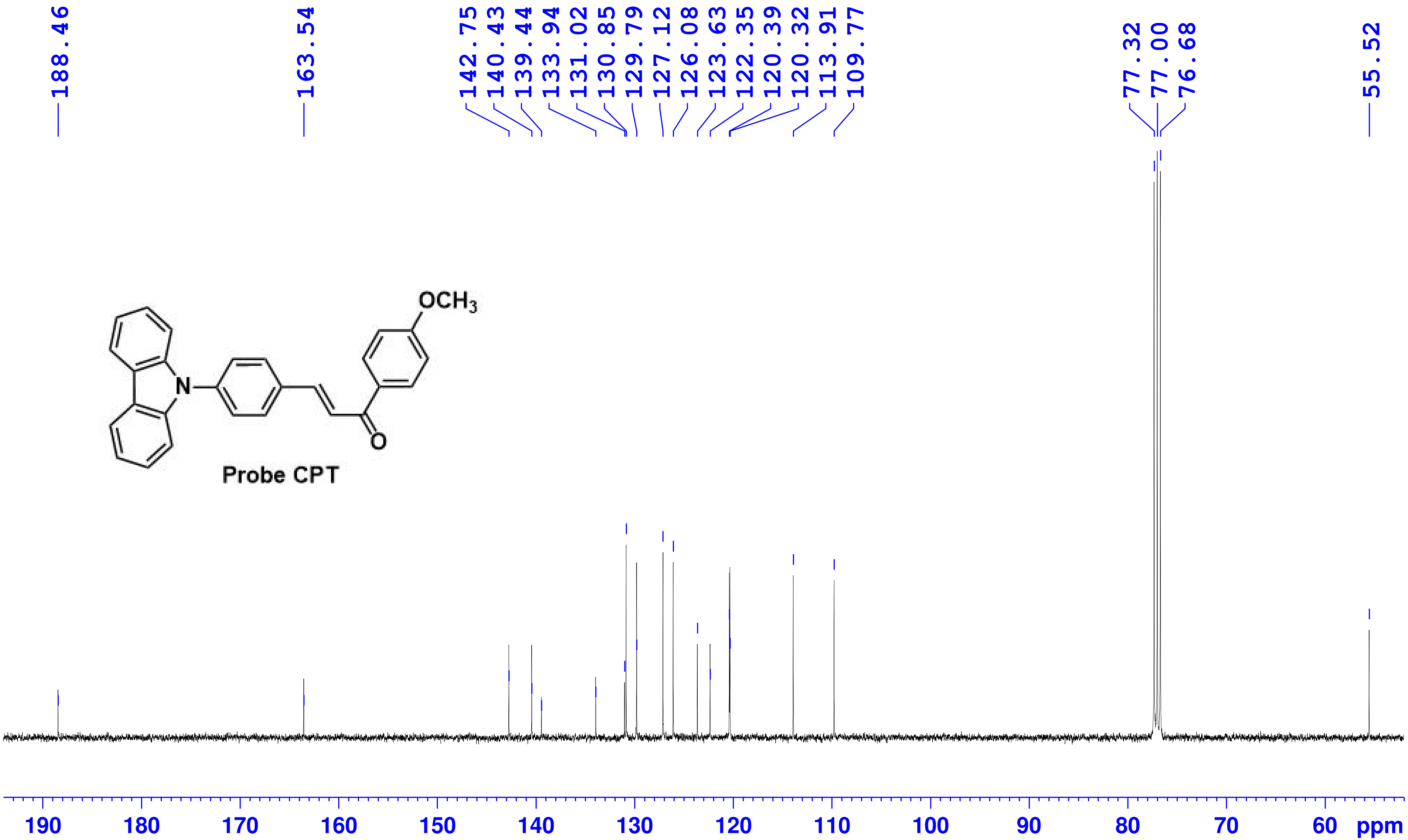

CPT Probe: 13C NMR (100 MHz, CDCl3)

HK-03/CDCl3/Huda Khalid/Dr. Umar Farooq CUI ATD(17-4-2025)

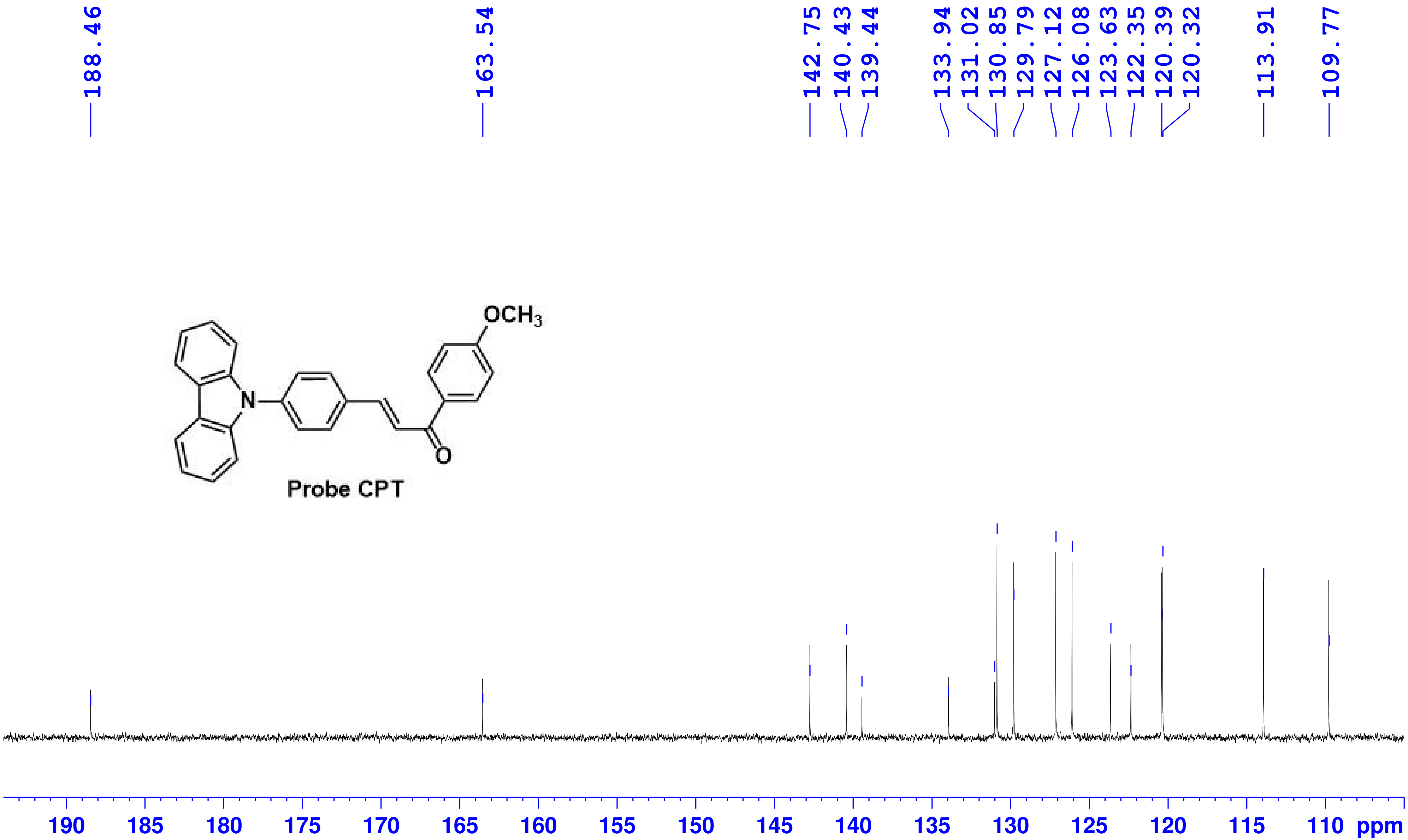

CPT Probe: 13C NMR (100 MHz, CDCl3)

HK-03/CDCl3/Huda Khalid/Dr. Umar Farooq CUI ATD(17-4-2025)

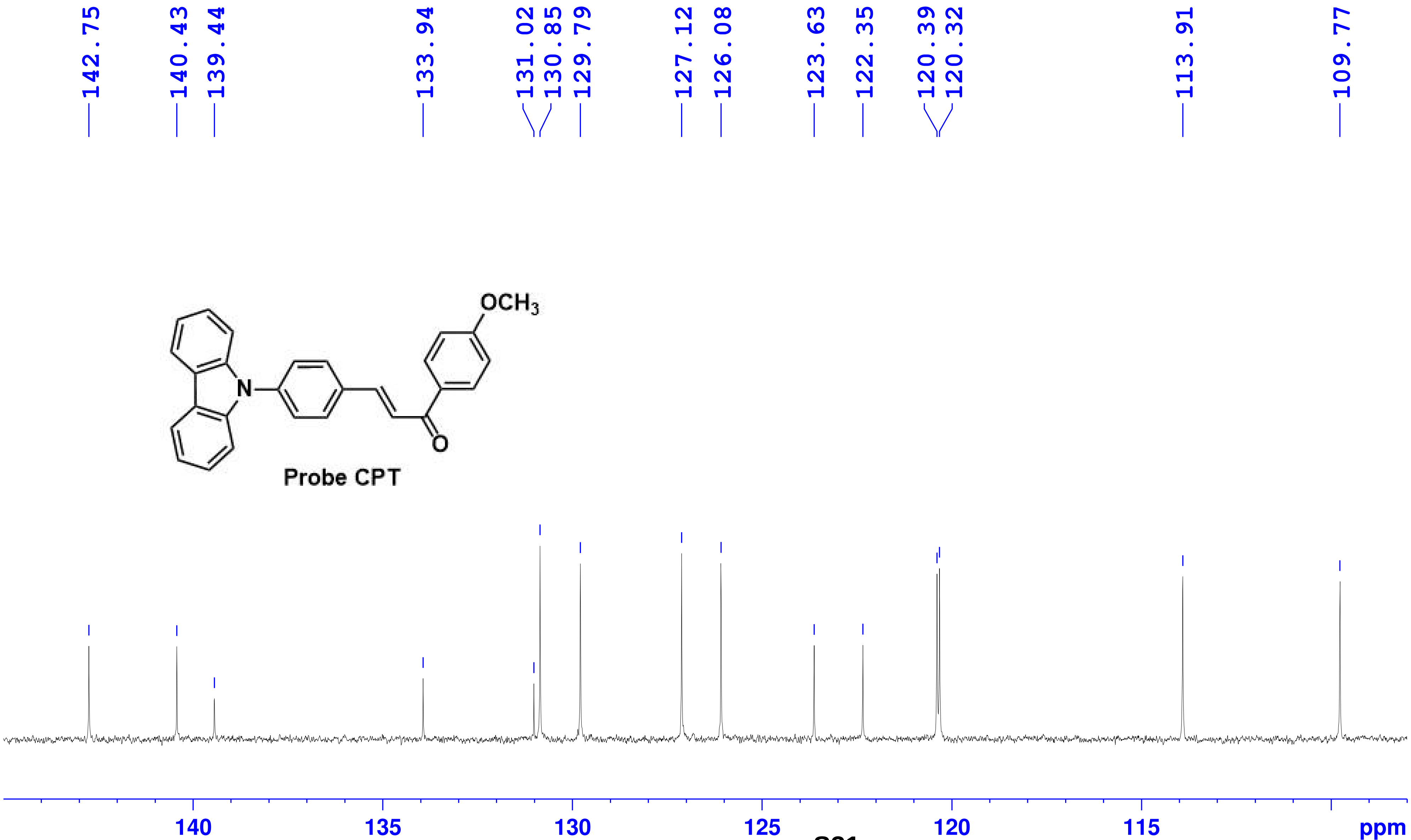

1H NMR Titration Experiment of Probe CPT + 4-Nitrotoluene

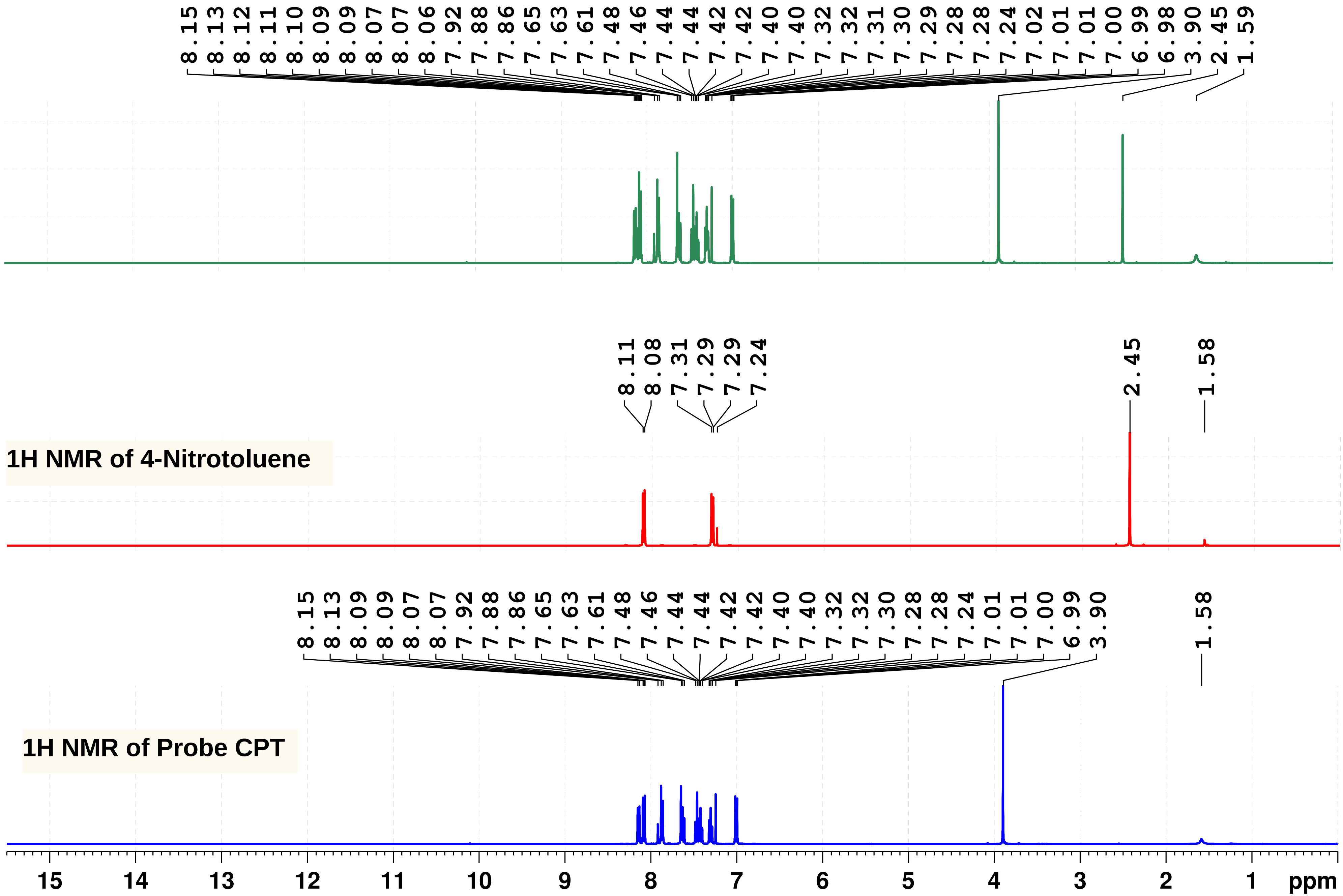

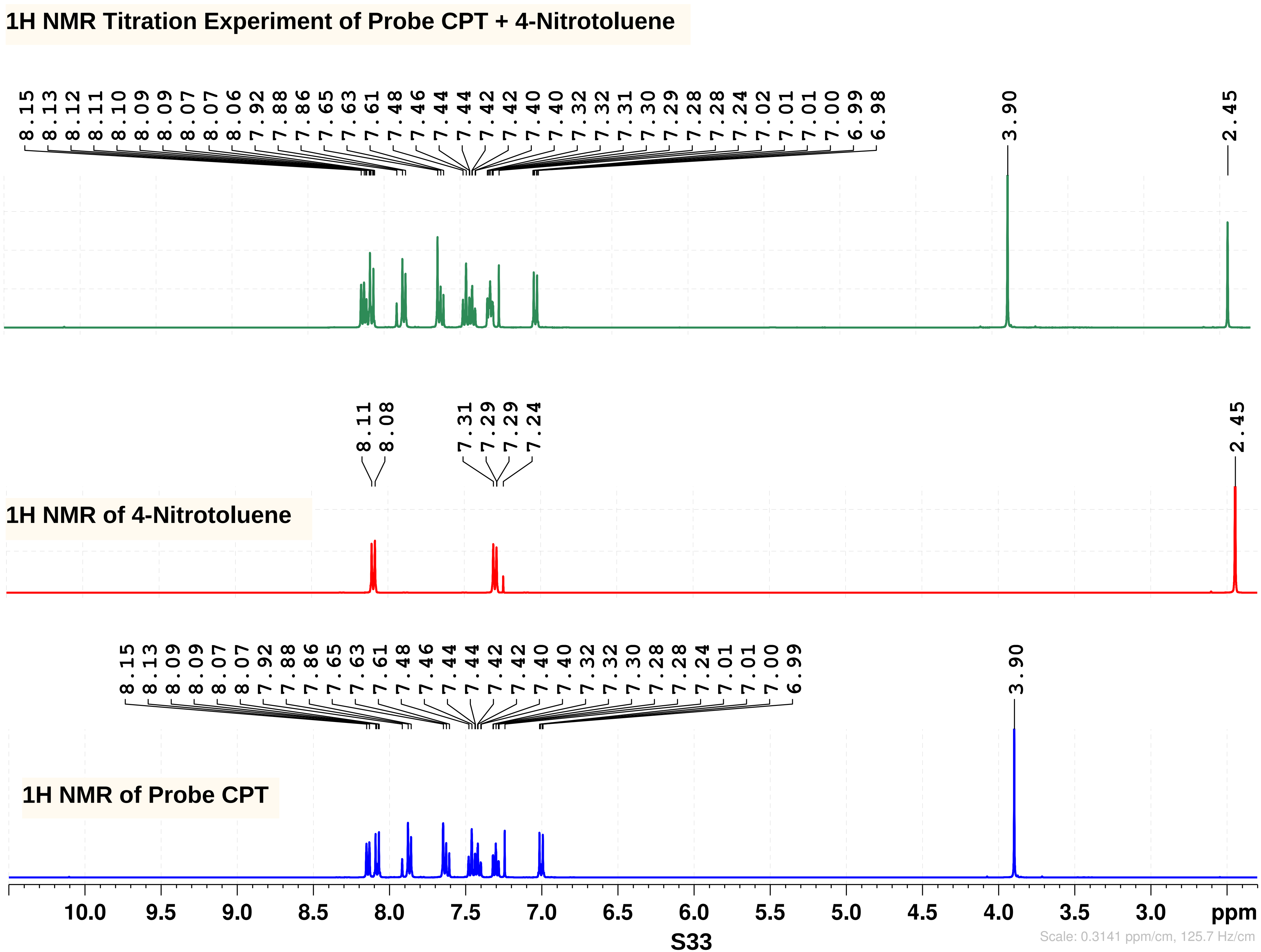
1H NMR Titration Experiment of Probe CPT + 4-Nitrotoluene
8.15 8.13 8.12 8.11 8.10 8.09 8.09 8.07 8.07 8.06 7.92 7.88 7.86 7.65 7.63 7.61 7.48 7.46 7.44 7.44 7.42 7.42 7.40 7.40 7.32 7.32 7.31 7.30 7.29 7.28 7.28 7.24 7.02 7.01 7.01 7.00 6.99 6.98
3.90
2.45
8.11 8.08
7.31 7.29 7.29 7.24
2.45
1H NMR of 4-Nitrotoluene
8.15 8.13 8.09 8.09 8.07 8.07 7.92 7.88 7.86 7.65 7.63 7.61 7.48 7.46 7.44 7.44 7.42 7.42 7.40 7.40 7.32 7.32 7.30 7.28 7.28 7.24 7.01 7.01 7.00 6.99
3.90
1H NMR of Probe CPT
10.0 9.5 9.0 8.5 8.0 7.5 7.0 6.5 6.0 5.5 5.0 4.5 4.0 3.5 3.0 ppm
Scale: 0.3141 ppm/cm, 125.7 Hz/cm

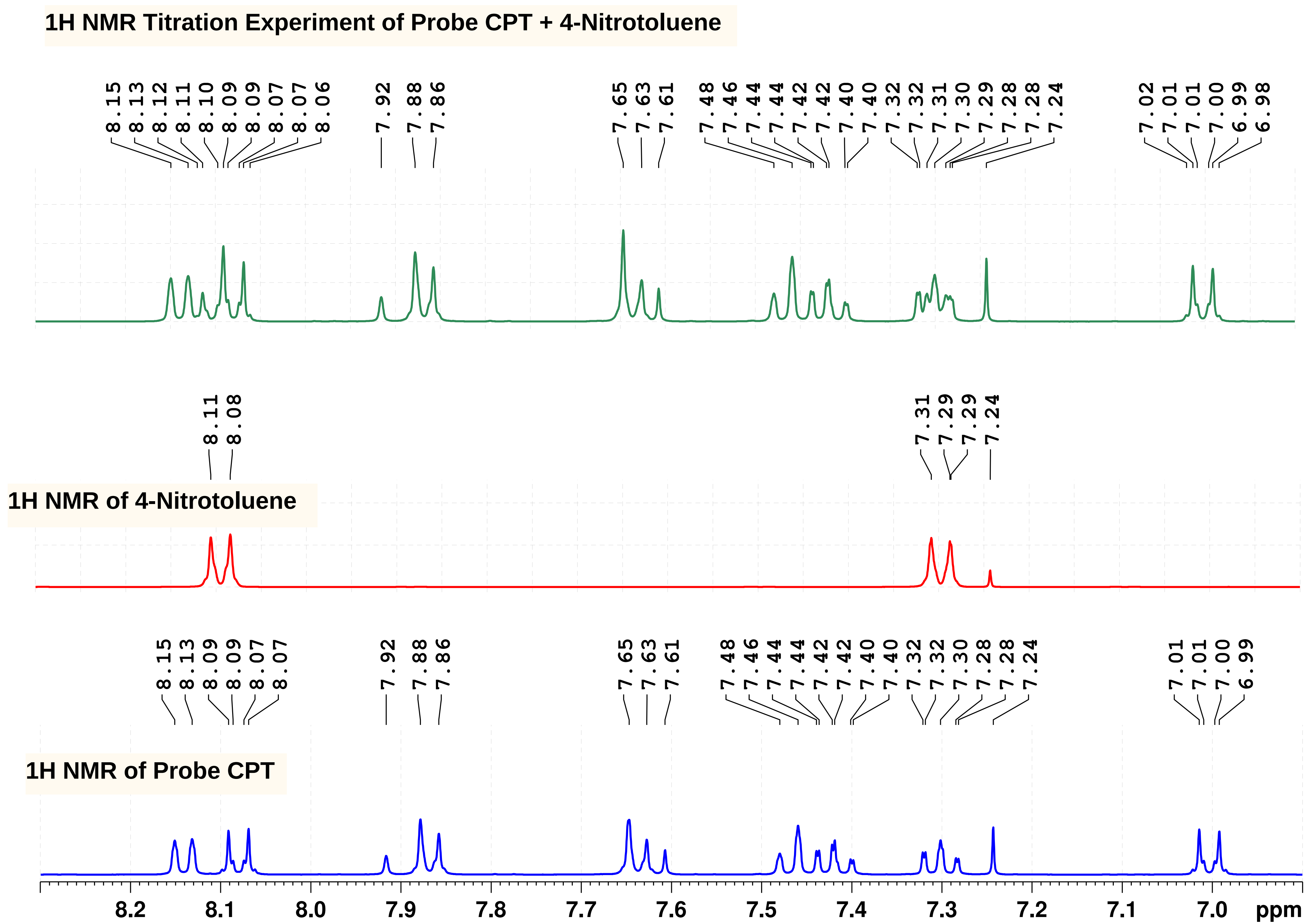
1H NMR Titration Experiment of Probe CPT + 4-Nitrotoluene
8.15 8.13 8.12 8.11 8.10 8.09 8.09 8.07 8.07 8.06 7.92 7.88 7.86 7.65 7.63 7.61 7.48 7.46 7.44 7.44 7.42 7.42 7.40 7.40 7.32 7.32 7.31 7.30 7.29 7.28 7.28 7.24 7.02 7.01 7.01 7.00 6.99 6.98
8.11 8.08 7.31 7.29 7.29 7.24
1H NMR of 4-Nitrotoluene
8.15 8.13 8.09 8.09 8.07 8.07 7.92 7.88 7.86 7.65 7.63 7.61 7.48 7.46 7.44 7.44 7.42 7.42 7.40 7.40 7.32 7.32 7.30 7.28 7.28 7.24 7.01 7.01 7.00 6.99
1H NMR of Probe CPT
8.2 8.1 8.0 7.9 7.8 7.7 7.6 7.5 7.4 7.3 7.2 7.1 7.0 ppm
Scale: 0.05362 ppm/cm, 21.46 Hz/cm
